# Targeting the RNA-binding motif protein 15 suppresses prostate cancer progression and hormone therapy resistance by promoting androgen receptor degradation

**DOI:** 10.1186/s43556-026-00428-1

**Published:** 2026-04-08

**Authors:** Bintao Hu, Le Li, Zhenghui Jin, Qinyu Li, Yue Wu, Jie Chen, Jihong Liu, Chenglin Han, Tao Wang

**Affiliations:** 1 Department of Urology, Tongji Hospital, Tongji Medical College, Huazhong University of Science and Technology, Wuhan, 430030 China; 2 Department of Urology, Jiangxi Provincial People’s Hospital, The First Affiliated Hospital of Nanchang Medical College, Nanchang, 330000 China; 3 Department of Urology, The First Affiliated Hospital of Shandong First Medical University & Shandong Provincial Qianfoshan Hospital, Jinan, 250000 China

**Keywords:** Prostate cancer, M^6^A, RBM15, AR, DDB1, YTHDF2

## Abstract

Androgen deprivation therapy (ADT) remains the standard treatment for advanced prostate cancer (PCa); however, most patients ultimately progress to lethal castration-resistant PCa (CRPC). Emerging evidence implicates RNA N⁶-methyladenosine (m⁶A) modification as a key regulator of cancer biology, yet its role in CRPC remains poorly understood. As a critical adaptor in the m⁶A methyltransferase complex, RNA-binding motif protein 15 (RBM15) directs m⁶A deposition to specific mRNA targets. Here, we identified RBM15 as the key methyltransferase member significantly upregulated in CRPC tissues and strongly correlated with poor patient survival. Functionally, RBM15 overexpression reduces PCa cell sensitivity to enzalutamide, whereas its knockdown suppresses tumor growth and invasion. Mechanistically, RBM15 is an androgen-responsive protein whose expression increases upon chronic androgen deprivation. It catalyzes m⁶A methylation at position A1384 of damaged DNA binding protein 1 (*DDB1*) mRNA, leading to YTHDF2-dependent transcript decay and reduced DDB1 protein levels. Lower DDB1 impairs K48-linked polyubiquitination of the androgen receptor (AR), thereby stabilizing AR and amplifying AR signaling. Importantly, AR transcriptionally activates *RBM15*, forming a feed-forward loop that drives CRPC progression. Collectively, our findings establish RBM15 as a central epitranscriptomic driver of CRPC and identify the RBM15–DDB1–AR axis as a promising therapeutic target. Dual inhibition of RBM15 and AR may offer a novel strategy to overcome treatment resistance in advanced PCa.

**Supplementary Information:**

The online version contains supplementary material available at 10.1186/s43556-026-00428-1.

## Introduction

Prostate cancer (PCa) remains the most prevalent malignancy in the male urogenital system, ranking second in global cancer incidence and fifth in cancer-related mortality among men [1]. As a hormone-driven disease, PCa progression depends heavily on androgen receptor (AR) signaling—making androgen deprivation therapy (ADT) the cornerstone of treatment for advanced disease [2]. Yet despite initial responses, nearly all patients relapse within 2–3 years and develop castration-resistant PCa (CRPC), with a 5-year survival rate of only ~ 30% in metastatic cases [3, 4]. This clinical challenge underscores an urgent need to uncover novel molecular mechanisms underlying therapy resistance.

N6-methyladenosine (m6A), the most abundant posttranscriptional RNA modification in eukaryotes, regulates RNA fate through a dynamic process orchestrated by "writers," "erasers," and "readers" [5–7]. These m6A collectively govern RNA metabolism, including splicing, stability, translation, and degradation [8–10]. Dysregulation of this m6A machinery is increasingly implicated in tumorigenesis, influencing oncogene expression, tumor microenvironment, and therapeutic resistance [11, 12]. While the involvement of m6A in cancers like lung, gastric, and liver malignancies has been documented [13–15], its role in PCa, particularly in the context of CRPC and therapy resistance, remains inadequately characterized.

Among the m⁶A writer complex, RNA-binding motif protein 15 (RBM15) serves not as a catalytic subunit but as a sequence-specific adaptor that recruits the core methyltransferase METTL3–METTL14 to target RNAs [16, 17]. Physiologically, RBM15 is indispensable for normal hematopoiesis and megakaryocyte differentiation, and its dysregulation is a recognized driver of leukemogenesis [16]. More recently, aberrant RBM15 expression has been observed in several solid tumors, hinting at a broader oncogenic potential [18–20]. However, its precise functional contribution and molecular mechanisms in these cancers—most notably in prostate cancer—remain largely uncharted territory. This knowledge gap is particularly significant given the emerging importance of m6A in cancer progression and resistance.

Considering the established central role of AR signaling in PCa and the critical function of m6A in regulating gene expression, we hypothesized that adaptor proteins like RBM15 could be a crucial link between the m6A machinery and AR-driven oncogenesis, especially in the transition to CRPC. Therefore, this study aims to define the role and underlying mechanisms of RBM15 in PCa progression and enzalutamide resistance.

Our study identified RBM15 as a pivotal factor strongly associated with PCa progression and poor prognosis. Through integrated biological experiments, we demonstrated that RBM15 promotes PCa tumorigenesis and enzalutamide resistance by modulating damaged DNA binding protein 1 (DDB1) signaling. Specifically, we proposed that targeting the RBM15-AR axis represents a novel strategy to overcome hormone therapy resistance. Our findings established RBM15 as a mechanistic driver of CRPC and a promising therapeutic target, providing new insights for clinical intervention in advanced PCa.

## Results

### RBM15 promotes PCa progression and growth, migration, invasion of PCa cells

To investigate the potential roles of RBM15 in patients with PCa, two-sample mendelian randomization (MR) was employed to determine the causality between RBM15 and PCa. A significant causal relationship between RBM15 and PCa was observed (*p* = 0.008), with an odds ratio of 1.042 and a 95% confidence interval of 1.011–1.074, suggesting that RBM15 is a risk factor for PCa (Fig. S1a). The MR sensitivity analysis revealed no significant pleiotropy (Fig. S1b-c and Table S1) or heterogeneity (Table S2) in this causal effect, suggesting that the results of MR analysis are reliable.

**Fig. 1:**
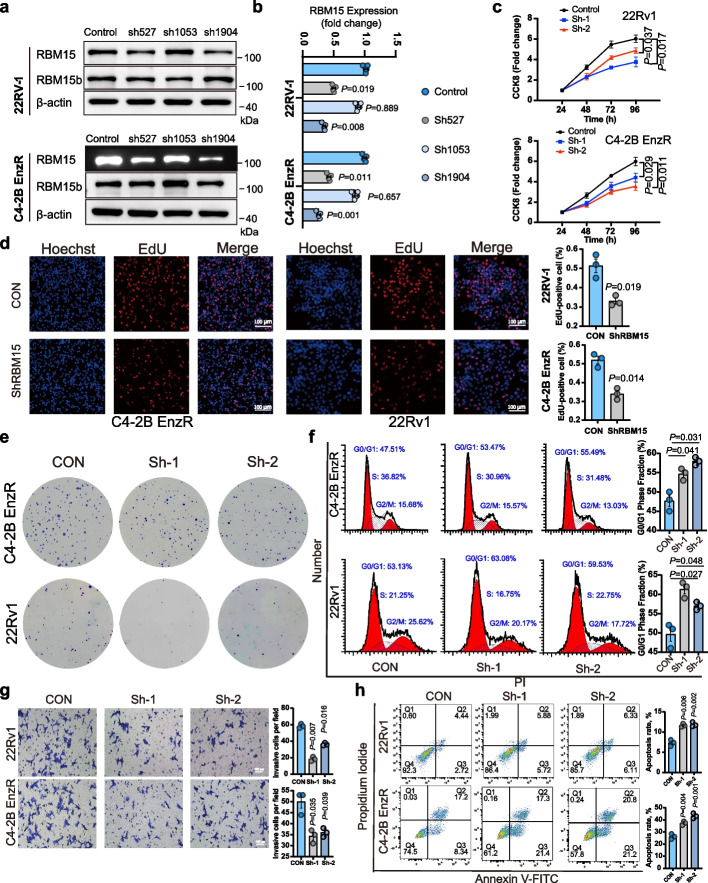
RBM15 silencing inhibits growth, migration, and invasion, as well as promotes apoptosis of PCa cells. **a**-**b** Representative (**a**) and statistical (**b**) analysis of RBM15 knockdown and RBM15b expression by lentivirus infection in PCa cells was evaluated using western blot (samples from 4 independent experiments). **c** CCK-8 experiments were employed to determine the changes in cell viability within 96 h following RBM15 knockdown. **d** Changes in the proliferative activity of C4-2B EnzR and 22Rv1 cells after RBM15 knockdown were examined by EdU assays (*n* = 3). **e** Colony formation assays were used to evaluate the impact of RBM15 silencing on the proliferative capacity of cells. **f** Flow cytometry was adopted to examine changes in various phases of the cell cycle following RBM15 knockdown (*n* = 3). **g** Changes in the invasion capabilities of C4-2B EnzR and 22Rv1 cells after RBM15 knockdown were assessed using transwell invasion assays (samples from 3 independent experiments). **h** The impact of RBM15 silencing on the apoptosis rate of PCa cells was evaluated by flow cytometry. The data are shown as mean ± SD. KD and CON represent RBM15 knockdown and the corresponding control group, respectively. SiNC denotes the negative control of siRNA. Two-tailed Student’s t test for **d** and One-way Anova test for **b**, **c**, **f**, **g**, **h**

To investigate RBM15's biological function in PCa, we generated stable RBM15 knockdown (ShRBM15) and overexpression (RBM15-OE) cell lines via lentiviral infection, including C4-2B EnzR (hormone resistant) and 22RV-1 (hormone sensitive) cells based on prior expression profiling [21]. We confirmed efficient RBM15 knockdown by sh527 (Sh-1), sh1904 (Sh-2) and verified successful overexpression rather than RBM15b in these cells (Fig. 1a-b). Functional assays revealed that ShRBM15 reduced cell viability, proliferation (Fig. 1c-d), and colony formation (Fig. 1e), while promoting G1 phase arrest (Fig. 1f). Conversely, RBM15-OE enhanced these pro-tumorigenic properties (Fig. S2a-e). Additionally, RBM15 knockdown significantly reduced migration and invasion capabilities of PCa cells and promoted their apoptosis (Fig. 1g, h), whereas RBM15 overexpression significantly increased their migration (Fig. S2f-i).

**Fig. 2:**
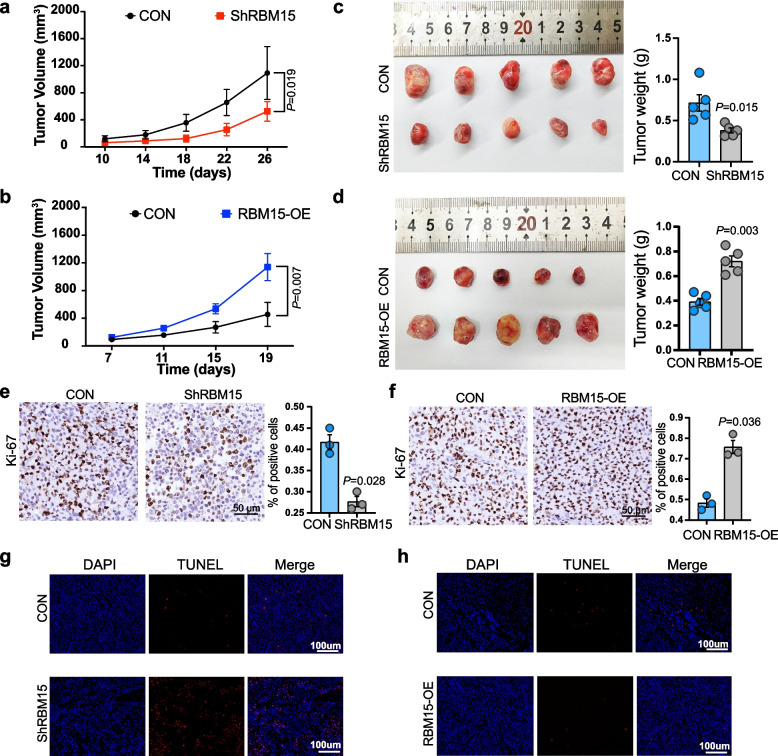
RBM15 affects the growth and apoptosis of xenograft tumors derived from PCa cells in vivo. **a**-**b** Growth curves of subcutaneous xenografts (*n* = 5 mice for each group) show the effect of RBM15 knockdown (**a**) or overexpression (**b**) on tumor growth in vivo. **c**-**d** Xenograft tumors with either knocked down (**c**) or overexpressed RBM15 (**d**) were removed from nude mice. Tumors were then photographed, weighed, and compared. **e**–**f** Immunohistochemical staining assays show the impact of RBM15 knockdown (**e**) or overexpression (**f**) on Ki-67-positive rates in tumors (samples from 3 independent experiments). **g**-**h** TUNEL assay was employed to detect apoptosis in xenograft tumors (samples from 3 independent experiments) following RBM15 knockdown (**g**) or overexpression (**h**). ShRBM15, RBM15-OE and CON represent RBM15 knockdown, RBM15 overexpression, and the corresponding control group, respectively. Scramble and vector serve as the negative controls for the knockdown and overexpression of RBM15 groups, respectively. All data are shown mean ± SD. One-way Anova test for **a**, **b**.Two-tailed Student’s t test for **c**,** d**,** e**,** f**

To validate RBM15 function in vivo, 22Rv1 cells with RBM15 overexpression and C4-2B EnzR cells with knockdown, along with controls, were subcutaneously implanted into nude mice. RBM15 knockdown suppressed tumor growth (Fig. 2a and c), while overexpression accelerated it (Fig. 2b and d). Immunohistochemistry results showed that RBM15 levels were positively correlated with Ki-67 expression (Fig. 2e-f), and TUNEL assays confirmed that RBM15 knockdown promoted cell apoptosis in tumor tissues (Fig. 2g-h).

### RBM15 promotes resistance to enzalutamide in PCa cells

**Fig. 3:**
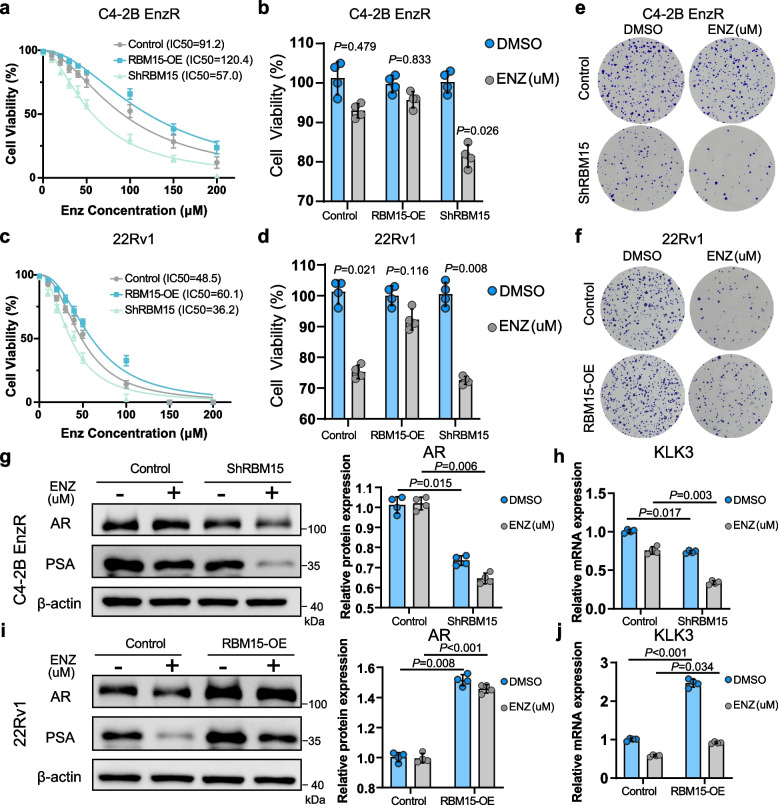
RBM15 promotes resistance to enzalutamide in PCa cells. **a**-**b** The effect of different concentrations of enzalutamide on the viability of C4-2B EnzR cells with RBM15 knockdown or overexpression. **c**-**d** Difference in the effect of enzalutamide on 22RV-1 cell viability between cells with and without RBM15 knockdown or overexpression (samples from 4 independent experiments). **e**–**f** Colony formation assays evaluating the impact of knocking down (**e**) or overexpressing (**f**). **g**-**j** The effect of knocking down (**g**,** h**) or overexpressing (**i**,** j**) RBM15, combined with or without enzalutamide treatment, on the expression of AR and its downstream target PSA (samples from 4 independent experiments). Cells were treated with enzalutamide for 3 days. ns denotes no significance. Enz denotes enzalutamide. KD and OE represent RBM15 knockdown and overexpression, respectively. Scramble and vector serve as the negative controls for the knockdown and overexpression of RBM15 groups, respectively. Data are displayed as mean ± SD. Two-tailed Student’s t test

To experimentally validate the link between RBM15 and enzalutamide sensitivity, we treated C4-2B Enzalutamide-resistant (EnzR) cells with a range of enzalutamide concentrations (0–200 μM). Lower RBM15 expression sensitized the cells to enzalutamide, whereas higher expression promoted resistance (Fig. 3a-d). Furthermore, colony formation assays assessing the effect of RBM15 on long-term drug response showed that RBM15 reduced inhibitory effects of enzalutamide on tumor cell migration (Fig. 3e-f). Apoptosis analysis corroborated these findings, demonstrating that RBM15 knockdown enhanced enzalutamide-induced apoptosis, while overexpression suppressed it (Fig. S3a-d). To uncover the underlying mechanism, we examined the AR signaling pathway and found that RBM15 knockdown reduced the expression of AR and its downstream target PSA, and potentiated the suppressive effect of enzalutamide on PSA levels (Fig. 3g-j). In summary, these findings provide strong evidence that RBM15 promotes enzalutamide resistance in prostate cancer by activating the AR signaling pathway.

Previous drug sensitivity analysis suggested RBM15 association with AMG-232 (p53-MDM2 inhibitor) [22], implicating potential p53 pathway involvement. To elucidate the molecular mechanisms downstream of RBM15, we investigated its impact on key cellular processes. Based on our previous analysis suggesting a link to the p53 pathway, we examined the expression of its core components. Western blot analysis showed that RBM15 knockdown led to the upregulation of p53 and its downstream effector p21, along with the downregulation of cyclin D1, a G1/S phase regulator (Fig. S3e). We then assessed the role of RBM15 in epithelial-mesenchymal transition (EMT). Consequently, RBM15 silencing resulted in decreased N-cadherin and increased E-cadherin, indicating an inhibition of EMT and suggesting a role for RBM15 in regulating cell migration and invasion (Fig. S3f). Concurrently, we found that RBM15 knockdown promoted the intrinsic apoptotic pathway by increasing the BAX/BCL2 ratio, as shown by the levels of these proteins in vitro and in vivo, respectively (Figs. S3g-i and S4a). Together, these results demonstrate that RBM15 exerts its oncogenic effects by simultaneously modulating the p53 cell cycle checkpoint, inhibiting EMT, and suppressing apoptosis.

### RBM15 facilitates the malignant biological behaviors of PCa cells by increasing AR protein expression

**Fig. 4:**
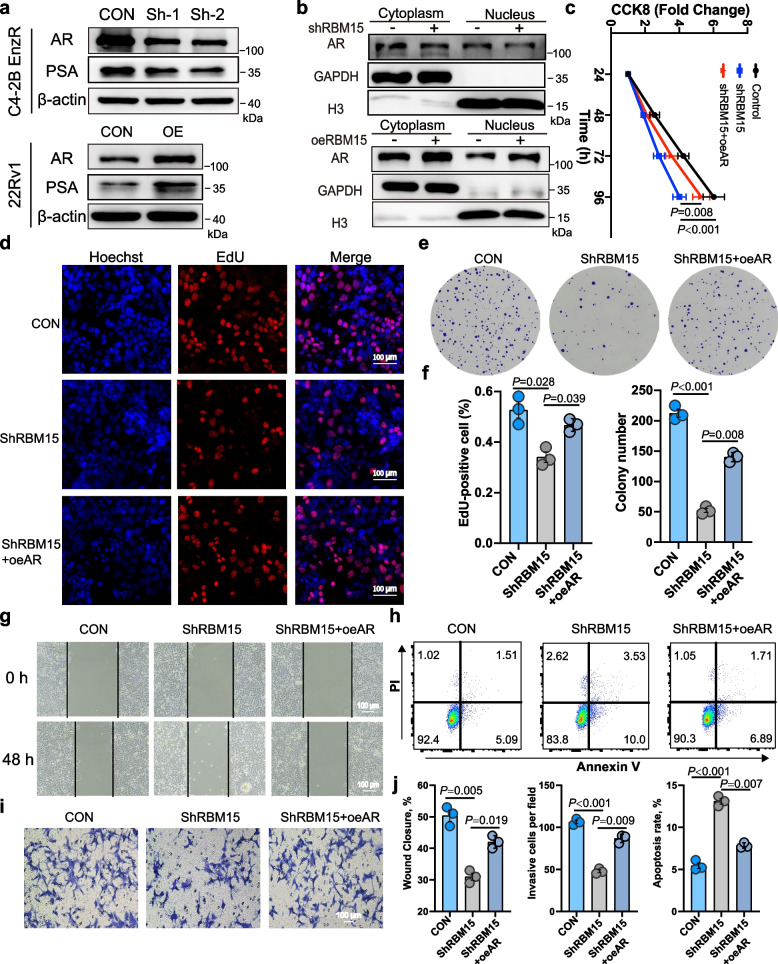
RBM15 facilitates the malignant biological behaviors of PCa cells by increasing AR protein expression. **a**-**b** Western blots showing the impact of RBM15 knockdown or overexpression on the expression of total AR, PSA, nuclear and cytoplasmic AR. **c** CCK-8 assays indicated that AR reversed the inhibitory effect of RBM15 silencing on cell viability (*n* = 3). **d** EdU experiments were used to assess the effects of RBM15 knockdown alone or in combination with AR overexpression on cell proliferation. **e** Representative images showing colony formation numbers under different treatments, including RBM15 knockdown alone or in combination with AR overexpression. **f** Statistical analysis for the results of the above EdU and colony formation assays (samples from 3 independent experiments). **g** Wound healing assays displaying the status of scratch closure at 48 h under different treatment conditions. **h** Flow cytometry for apoptosis showing the apoptosis rate under different treatment conditions. **i** Transwell invasion experiments exhibiting the number of invasive cells under treatment of RBM15 knockdown alone or in combination with AR overexpression. **j** Statistical analysis for the results of wound healing, transwell invasion, and apoptosis assays (samples from 3 independent experiments). All data are presented as mean ± SD. KD, OE, and CON represent RBM15 knockdown, RBM15 overexpression, and the corresponding control group, respectively. One-way Anova test

To verify RBM15's regulation of AR activity, we first examined its effect on AR and PSA expression. RT-qPCR showed that RBM15 significantly regulated PSA mRNA expression (Fig. 3h and j). Moreover, Western blotting revealed that RBM15 regulated both AR and PSA protein levels (Figs. 3g, i and 4a). Further analysis indicated that RBM15 significantly increased both nuclear and plasmic AR expression (Fig. 4b), suggesting a post-transcriptional or post-translational regulatory mechanism.

To elucidate whether AR mediates RBM15's effects on PCa cell behavior, rescue experiments were performed in C4-2B enzalutamide-resistant (EnzR) cells. Overexpression of AR substantially reversed the suppressive effects of RBM15 silencing on cell viability, proliferation, and clonogenic potential (Fig. 4c-f). Moreover, AR overexpression counteracted the RBM15 knockdown-induced reduction in cell migration and invasion, as well as the elevation in apoptosis rate (Fig. 4g-j). Together, these results suggest that RBM15 promotes PCa progression predominantly through modulating AR expression and activity.

### RBM15 inhibits AR degradation through the ubiquitin–proteasome pathway

**Fig. 5:**
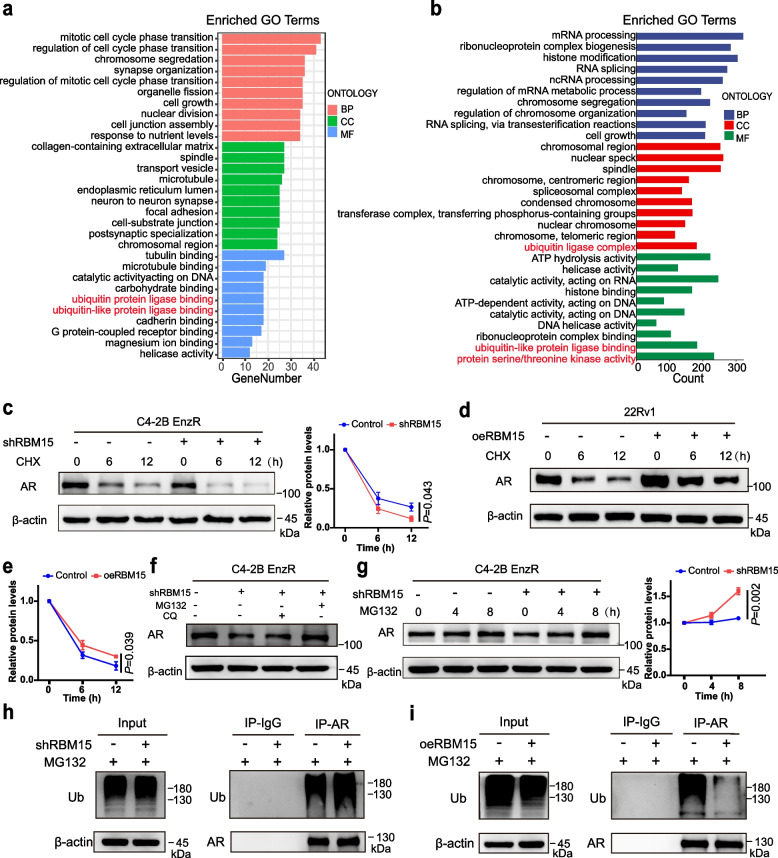
RBM15 inhibits AR degradation through the ubiquitin–proteasome pathway. **a** GO enrichment analysis was performed on significantly differentially expressed genes caused by RBM15 overexpression in RNA sequencing data. **b** In the GSE73893 dataset, GO enrichment analyses were conducted on the RNA bound by RBM15 protein. **c** The impact of RBM15 knockdown on AR protein degradation under various exposure times to 20 μg/mL cycloheximide (*n* = 3). **d**-**e** The impact of RBM15 overexpression on AR protein degradation under various exposure times to 20 μg/mL cycloheximide in 22Rv1 cells (*n* = 3). **f** C4-2B EnzR cells were knocked down by shRBM15 lentivirus and then treated with 10 μM KG132 or chloroquine for 12 h. The expression of AR proteins was measured using western blotting. **g** Changes in AR protein expression were observed after 0, 4, 8 h of MG132 treatment in C4-2B EnzR cells, following lentiviral-mediated RBM15 knockdown (*n* = 3). **h**-**i** Coimmunoprecipitation experiments revealing the effects of RBM15 knockdown (**h**) or overexpression (**i**) on the ubiquitination levels of AR proteins. Data are shown mean ± SD. CHX represents cycloheximide. CQ denotes chloroquine. One-way Anova test

To elucidate the mechanisms by which RBM15 regulates androgen receptor (AR), we performed RNA sequencing on RBM15-overexpressing 22Rv1 cells to identify potential downstream pathways. Gene Ontology (GO) enrichment analysis of the sequencing data indicated that RBM15 is involved in the regulation of the cell cycle, cellular growth, and ubiquitin ligase binding (Fig. 5a), which shows the significantly enriched biological processes and molecular functions. Considering the established function of RBM15 as an RNA-binding protein, we further re-analyzed publicly available RBM15 RNA immunoprecipitation sequencing (RIP-seq) data (GSE73893), which revealed a significant enrichment of RBM15-bound transcripts associated with ubiquitin ligase complexes and mRNA metabolic processing (Fig. 5b). Given our previous finding that RBM15 regulates AR protein but not its mRNA levels, we hypothesized that RBM15 modulates AR expression through the ubiquitin–proteasome system. To validate this hypothesis, we conducted cycloheximide (CHX) chase assays to assess AR protein stability. These assays demonstrated that RBM15 knockdown accelerated the degradation rate of AR protein, while RBM15 overexpression had the opposite effect, stabilizing the protein, as shown by the time-course Western blots and corresponding quantification of AR half-life (Fig. 5c-e). To pinpoint the specific degradation pathway, RBM15-depleted cells were treated with either the proteasome inhibitor MG132 or the lysosomal inhibitor chloroquine (CQ). The results revealed that treatment with MG132, but not CQ, successfully rescued the RBM15 knockdown-induced reduction in AR protein levels (Fig. 5f-g). This confirmed that RBM15 regulates AR protein via the ubiquitin–proteasome pathway. Furthermore, immunoprecipitation (IP) assays were performed to directly measure AR ubiquitination, which showed that RBM15 knockdown markedly increased the ubiquitination of AR, whereas RBM15 overexpression significantly decreased it (Fig. 5h-i). Collectively, these findings demonstrate that RBM15 enhances AR protein stability by suppressing its ubiquitination and subsequent proteasomal degradation.

### RBM15 regulates the DDB1-mediated K48-linked polyubiquitination of AR proteins

**Fig. 6:**
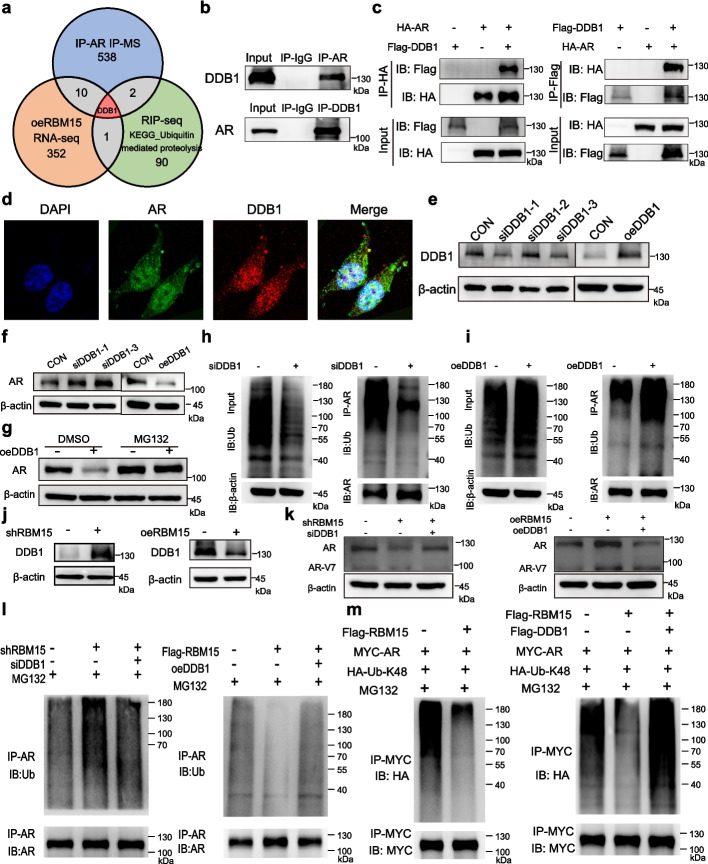
RBM15 upregulates AR expression by decreasing K48-linked polyubiquitination levels of AR via DDB1. **a** Integrative analysis of the mass spectrometry of coimmunoprecipitation with anti-AR antibody, RBM15-overexpressing RNA-seq, and RBM15 RIP-seq data to identify molecules that mediate RBM15’s regulation of AR. **b** Coimmunoprecipitation experiments were conducted to detect the protein interaction between AR and DDB1 in 22Rv1 cells. **c** HA-tagged AR and Flag-tagged DDB1 plasmids were transfected into 293 T cells for 72 h, followed by immunoprecipitation experiments to assess the protein interaction between DDB1 and AR. **d** Images captured by confocal microscopy showing the cellular distribution of AR and DDB1 proteins in 22Rv1 cells. **e** Western blotting was employed to evaluate the efficiency of DDB1 knockdown in 22Rv1 cells via siRNA and DDB1 overexpression in C4-2B EnzR cells using a plasmid transfection. **f** The impact of silencing and overexpressing DDB1 on AR protein expression in 22Rv1 and C4-2B EnzR cells, respectively. **g** Alterations of AR protein expression in C4-2B EnzR cells due to DDB1 overexpression alone or in combination with MG132 treatment. **h**-**i** Immunoprecipitation assays assessed the effect of knocking down (**h**) and overexpressing (**i**) DDB1 on the ubiquitination levels of AR proteins in 22Rv1 and C4-2B EnzR cells, respectively. **j** Western blotting showing that RBM15 could regulate DDB1 protein expression in C4-2B EnzR and 22Rv1 cells. **k** Rescue assays exhibiting that DDB1 could reverse the RBM15-induced alterations of AR and AR-V7 proteins in C4-2B EnzR and 22Rv1 cells. **l** Immunoprecipitation assays showing that DDB1 reversed the RBM15-induced changes in ubiquitination levels of AR proteins in C4-2B EnzR and 22Rv1 cells. **m** Western blotting demonstrated that RBM15 regulated the K48-linked polyubiquitination levels of AR in 293 T cells, which could be reversed by DDB1. CON denotes the control group

To identify the mediators responsible for RBM15-regulated AR ubiquitination, we employed an integrated analysis of mass spectrometry, RNA sequencing, and RIP-seq data, which identified damage-specific DNA binding protein 1 (DDB1) as a top candidate (Fig. 6a). Given that DDB1 is a known component of the Cullin4A-RING E3 ubiquitin ligase complex [23], we first investigated its interaction with AR. Co-immunoprecipitation and confocal microscopy experiments confirmed both the physical interaction and subcellular colocalization between DDB1 and AR (Fig. 6b-d). We then assessed the functional impact of DDB1 on AR protein stability using loss- and gain-of-function approaches. Following the validation of three distinct DDB1 siRNAs and a DDB1 overexpression plasmid (Fig. 6e), we observed that DDB1 knockdown significantly increased AR protein levels, whereas DDB1 overexpression had the opposite effect (Fig. 6f). This regulation was abrogated when cells were co-treated with the proteasome inhibitor MG132 (Fig. 6g), confirming that DDB1 modulates AR via the proteasome. Consistent with this, immunoprecipitation assays revealed that DDB1 knockdown markedly reduced AR ubiquitination, while its overexpression enhanced it (Fig. 6h-i), confirming that DDB1 functions as an E3 ubiquitin ligase component to promote AR degradation. To ascertain if DDB1 acts downstream of RBM15, we first examined the effect of RBM15 on DDB1 expression and found that RBM15 overexpression negatively regulated DDB1 protein levels (Fig. 6j). Subsequently, a rescue experiment demonstrated that co-overexpression of DDB1 effectively reversed the RBM15-induced increase in AR protein (Fig. 6k), positioning DDB1 as a critical downstream effector. We then investigated the epistatic relationship between RBM15 and DDB1 in the context of AR ubiquitination. The results showed that DDB1 overexpression successfully counteracted the inhibitory effect of RBM15 on total AR ubiquitination (Fig. 6l). Since K48-linked polyubiquitin chains are the canonical signal for proteasomal degradation, we specifically evaluated this modification. Our findings revealed that RBM15 significantly reduced K48-linked polyubiquitination of AR, and this effect was robustly reversed by DDB1 overexpression (Fig. 6m). In summary, these results confirm that RBM15 stabilizes AR protein by negatively regulating the expression of DDB1, which in turn diminishes DDB1-mediated K48-linked polyubiquitination of AR and prevents its proteasomal degradation.

### RBM15-mediated m6A modifications impair DDB1 mRNA stability in a YTHDF2-dependent manner

**Fig. 7:**
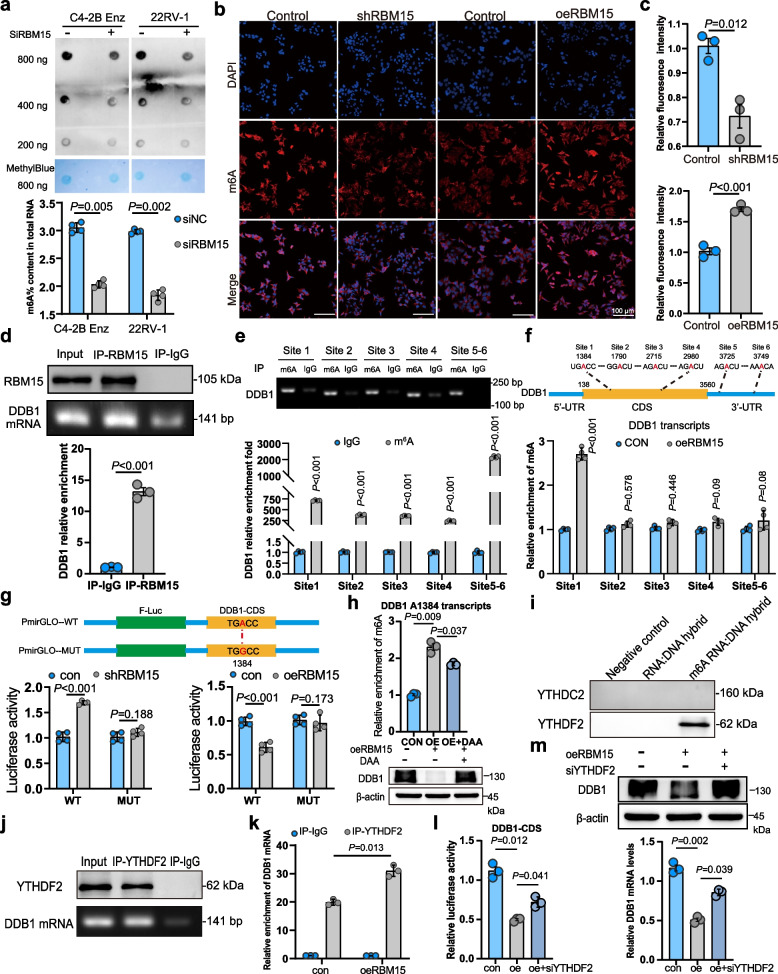
RBM15-mediated m6A modifications impair DDB1 mRNA stability in a YTHDF2-dependent manner. **a** Dot blotting and m6A RNA Methylation Quantification Kit showing the impact of RBM15 silencing on the levels of m6A modifications (*n* = 4). **b**-**c** Immunofluorescence experiments revealing the effect of knocking down or overexpressing RBM15 on intracellular m6A modification levels (*n* = 3). **d** RT‒qPCR experiments were conducted to detect changes in DDB1 mRNA following RBM15 knockdown or overexpression (*n* = 3). **e** m6A-modified sites in DDB1 mRNA were detected by Merip-qPCR and agarose electrophoresis analysis (*n* = 4). **f** Merip-qPCR experiments were conducted to investigate the impact of RBM15 on the m6A modification levels at various sites within DDB1 mRNA (*n* = 4). **g** The A1384 site within DDB1 CDS was mutated. A luciferase reporter assay was then conducted to examine the effects of RBM15 knockdown or overexpression, together with the mutation, on the luciferase activity of DDB1-CDS (*n* = 4). **h** Merip-qPCR and western blot experiments were conducted to assess the effects of overexpressing RBM15 alone or in combination with 50 μM 3-deazaadenosine on the alterations in m6A modification levels at the A1384 site and DDB1 protein expression (*n* = 3). **i** RNA pull-down assay was performed to identify the proteins bound by m6A-RNA: DNA hybrid probe. **j** RIP-qPCR with YTHDF2 antibody and agarose electrophoresis analysis confirmed the interaction between YTHDF2 proteins and DDB1 mRNA. **k** RIP-qPCR analysis showing the impact of RBM15 on the interaction between YTHDF2 proteins and DDB1 mRNA (*n* = 3). **l** Luciferase reporter assay demonstrated the role of YTHDF2 in the RBM15-induced reduction of DDB1-CDS luciferase activity (*n* = 3). **m** The impact of YTHDF2 and RBM15 on the expression of DDB1 mRNA and protein was investigated (*n* = 3). All data are presented as mean ± SD. KD, OE, and CON represent RBM15 knockdown, RBM15 overexpression, and the corresponding control group, respectively. WT and MUT represent wild type and mutation, respectively. DAA denotes 3-deazaadenosine. Two-tailed Student’s t test for **a**, **c**, **d**, **e**, **f**, **g**, **h**, **k**. One-way Anova test for **l**, **m**

RBM15 is an identified component of the m6A methyltransferase complex, which mediates m6A formation on cellular mRNAs [24]. Considering that RBM15 also regulates DDB1 protein expression, we hypothesized that RBM15 might modulate intracellular DDB1 levels via an m6A-dependent mechanism. To explore this hypothesis, we first investigated the effect of RBM15 on global m6A modification levels in cells. Dot blot assays and quantitative analysis revealed that RBM15 significantly altered cellular m6A modification levels (Fig. 7a). This was further corroborated by immunofluorescence microscopy, which showed that both RBM15 knockdown and overexpression markedly affected intracellular m6A levels (Fig. 7b-c and Fig. S5a-b).

Next, we investigated the underlying mechanisms by which RBM15 regulates DDB1 protein expression. RT-qPCR experiments demonstrated that RBM15 significantly affected DDB1 mRNA expression (Fig. 7d). To determine if this effect was transcriptional, dual-luciferase reporter assays were performed, which showed that RBM15 did not influence the transcriptional activity of the DDB1 promoter (Fig. S5c), thereby excluding transcriptional regulation. RNA stability assays subsequently revealed that RBM15 significantly impacted the degradation rate of DDB1 mRNA (Fig. S5d-e). Taken together, these results indicate that RBM15 regulates DDB1 levels in cells by affecting DDB1 mRNA stability.

Potential m6A modification sites within DDB1 mRNA were predicted using the SRAMP database and validated via MeRIP-qPCR. Six high-confidence sites identified by SRAMP were all confirmed to be m6A-modified (Fig. 7e). Notably, RBM15 specifically regulated the m6A modification level at the A1384 site (Fig. 7f). To assess the functional impact of this site, we performed site-directed mutagenesis and dual-luciferase reporter assays. RBM15 negatively regulated the luciferase activity of the wild-type A1384 construct but had no effect on the mutated version (Fig. 7g), indicating that RBM15 regulates DDB1 expression through m6A modification at this specific locus. Furthermore, treatment with 3-deazaadenosine (DAA), an m6A inhibitor, abolished the negative regulatory effect of RBM15 on DDB1 expression (Fig. 7h). Collectively, these findings demonstrate that RBM15 regulates DDB1 expression by modulating m6A modification at the A1384 site within DDB1 mRNA.

Building on previous findings that m6A reader proteins preferentially bind to methylated DNA:RNA hybrids [25], we designed biotin-labeled probes to identify m6A readers binding to the A1384 site on DDB1 mRNA. Given that YTHDF2 and YTHDC2 are primary m6A readers promoting mRNA degradation [8], we conducted RNA pulldown and RIP‒qPCR experiments to assess their binding to DDB1 mRNA. Our results revealed that YTHDF2, but not YTHDC2, bound to DDB1 mRNA (Fig. 7i), which was further confirmed by RIP‒qPCR (Fig. 7j-k).

To determine whether YTHDF2 mediates RBM15's regulation of DDB1, we performed siRNA-mediated knockdown of YTHDF2 and analyzed its effects on DDB1 expression. Western blot and RT‒qPCR analyses showed that YTHDF2 knockdown significantly increased DDB1 protein levels, with si-3 (siYTHDF2) exhibiting the strongest effect (Fig. S5f). This knockdown also inhibited DDB1 mRNA degradation and increased its expression (Fig. S5g). Dual-luciferase reporter assays demonstrated that silencing YTHDF2 reversed the RBM15-mediated reduction in DDB1-CDS luciferase activity (Fig. 7l). Furthermore, western blot and RT‒qPCR analyses indicated that YTHDF2 knockdown counteracted the RBM15-induced decrease in DDB1 expression (Fig. 7m). Collectively, these findings suggest that YTHDF2 facilitates RBM15-mediated regulation of DDB1 expression through m6A modification.

### RBM15 is regulated by the androgen signaling pathway

**Fig. 8:**
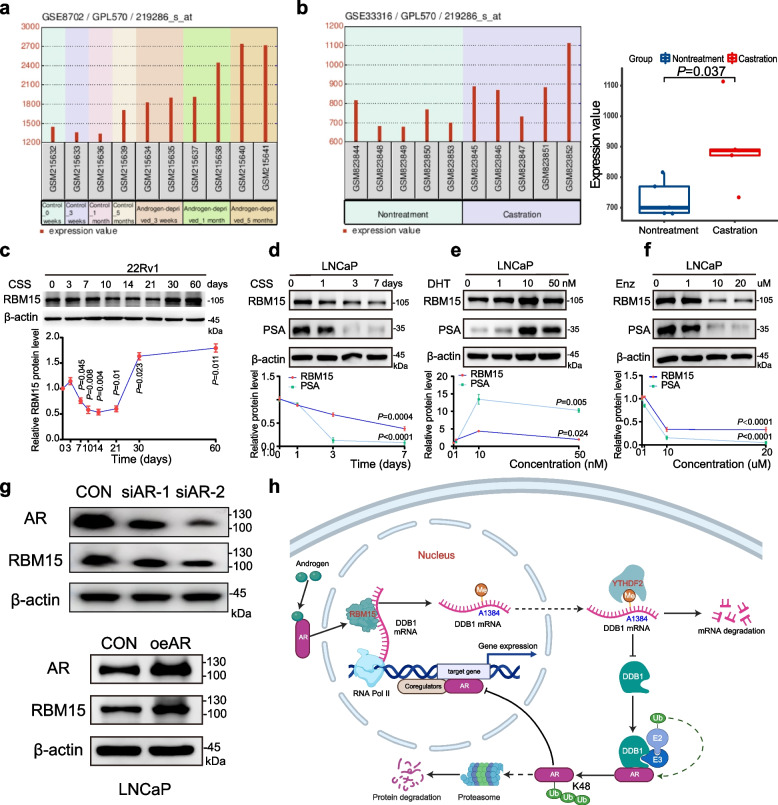
RBM15 is associated with castration-resistance progression and is regulated by the androgen signaling pathway. **a** GEO2R analysis of the GSE8702 dataset revealed changes in RBM15 expression in LNCaP cells during five months of androgen deprivation. **b** GEO2R analysis for GSE33316 datasets showed alterations of RBM15 expression in xenograft tumors four weeks post-surgical castration. **c** 22Rv1 cells were cultured in phenol red-free complete medium containing charcoal-stripped fetal bovine serum for two months, during which changes in RBM15 protein expression were monitored (*n* = 3). **d** The impact of short-term androgen deprivation on RBM15 and PSA proteins in LNCaP cells (*n* = 3). **e** LNCaP cells were pre-treated with androgen-free complete medium for 48 h, followed by treatment with various concentrations of dihydrotestosterone for an additional 24 h (*n* = 3). Changes in RBM15 and PSA proteins were then examined. **f** The effects of different concentrations of enzalutamide on the expression of RBM15 and PSA proteins were examined (*n* = 3). **g** The impact of AR knockdown and overexpression on RBM15 protein expression. **h** The proposed model illustrates the mechanism linking RBM15 and AR. CSS represents charcoal-stripped fetal bovine serum. DHT and Enz denote dihydrotestosterone and enzalutamide, respectively. Two-tailed Student’s t test for **b**. One-way Anova test for **c**, **d**,** e**,** f**

Analysis of the GSE74367 dataset revealed elevated RBM15 expression in castration-resistant prostate cancer (PCa) compared to primary PCa [21]. To investigate the relationship between castration and RBM15 expression, we examined the effects of androgen deprivation on RBM15 levels using multiple datasets. Analysis of the GSE8702 dataset demonstrated significant upregulation of RBM15 in LNCaP cells within three to five months following androgen deprivation (Fig. 8a). Similarly, data from the GSE33316 dataset revealed increased RBM15 expression in LuCaP35-derived xenografts four weeks post-surgical castration (Fig. 8b). To further validate these findings, we cultured 22Rv1 cells in charcoal-stripped fetal bovine serum (CS-FBS), a standard method for simulating androgen-deprived conditions in PCa research [26]. Our results showed that RBM15 expression initially decreased approximately ten days after androgen deprivation but subsequently increased substantially after one month (Fig. 8c). These findings suggest that androgen deprivation has a time-dependent effect on RBM15 expression, with short-term suppression followed by long-term enhancement.

To investigate whether the androgen signaling pathway regulates RBM15 expression, androgen-sensitive LNCaP cells were subjected to androgen deprivation or dihydrotestosterone (DHT) stimulation. Androgen deprivation significantly decreased RBM15 expression (Fig. 8d), whereas DHT supplementation following deprivation increased RBM15 levels (Fig. 8e), indicating that RBM15 is an androgen-responsive protein. Furthermore, treatment with enzalutamide, an androgen receptor inhibitor, significantly reduced RBM15 expression (Fig. 8f). The consistent alterations in PSA and RBM15 protein levels under androgen deprivation, DHT stimulation, or enzalutamide treatment suggest that androgen signaling positively regulates RBM15 expression. This was further corroborated by the observation that AR knockdown or overexpression significantly altered RBM15 expression (Fig. 8g).

Based on these findings, we propose a positive feedback regulatory loop involving RBM15 and AR (Fig. 8h).

## Discussion

Prostate cancer (PCa) is the most common male urogenital malignancy. While androgen deprivation therapy (ADT) is standard, most patients develop castration-resistant PCa (CRPC) within three years [27]. Current CRPC treatments, including abiraterone and enzalutamide, offer only limited progression-free survival [28]. This urgent clinical need underscores the importance of identifying novel molecular mechanisms to develop effective diagnostic, prognostic, and therapeutic strategies. Here, we identified RBM15 as the key factor being associated with both PCa progression and patient prognosis. RBM15, a component of the m6A methyltransferase complex [29], mediates m6A modifications by binding to target RNA. Its procarcinogenic role is well-established across various cancers. For example, RBM15 promotes proliferation, migration, and metastasis in pancreatic cancer [30], upregulates TMBIM6 in laryngeal squamous cell carcinoma [19], and drives tumor growth in triple-negative breast cancer [31]. These observations suggest a similar oncogenic function in PCa.

However, studies on RBM15 in PCa have been limited and contradictory. Our previous work and another study reported that RBM15 enhances CRPC cell proliferation, migration, stemness, and reduces apoptosis, acting as a prognostic biomarker [21]. Conversely, Ying et al. [25] found RBM15 inhibited PC-3 and DU-145 cell proliferation and migration via SEMA3F, while Zhang et al. [32] proposed a p53-mediated tumor-suppressive role. These discrepancies highlight a lack of definitive functional validation. Notably, Zhang et al.'s work primarily focused on molecular regulation without direct biological experiments on PCa cell malignant behaviors[32]. Our study resolves this ambiguity by providing comprehensive in vivo and in vitro evidence. We unequivocally demonstrate RBM15's oncogenic function in PCa, showing consistent changes in malignant behaviors and key protein expression (cell cycle, EMT, apoptosis) following RBM15 modulation. Multi-faceted bioinformatics analyses further support RBM15's role as an oncogene linked to PCa progression and poor prognosis.

Androgen receptor (AR) signaling remains critical in PCa progression, even at castration levels, which explains the transient efficacy of next-generation AR-targeted therapies in CRPC [2, 33]. Therefore, blocking AR signaling remains a primary therapeutic goal. Our study uncovers a novel mechanism: RBM15 upregulates AR protein expression by modulating its degradation via the ubiquitin–proteasome system (UPS), thereby promoting PCa malignant behaviors. This is highly relevant to CRPC, as we observed enhanced RBM15 expression during long-term castration, leading to increased AR expression. This dynamic aligns with the sustained AR dependency in advanced PCa. The UPS is a major protein degradation pathway [34], with K48-linked polyubiquitination being a common signal for proteasomal degradation [35, 36]. AR protein degradation, regulated by the UPS, is known to influence PCa progression [37, 38].

We further pinpointed the molecular details: RBM15 reduces K48 polyubiquitination levels of AR protein by downregulating DDB1 expression, consequently increasing AR protein levels. DDB1, a component of the Cul4A/B ubiquitin E3-ligase complex, targets substrates for ubiquitination [23, 39]. We identified a direct interaction between DDB1 and AR, which facilitates AR ubiquitination and degradation. This aligns with previous reports where PROTACs recruit DDB1 to degrade new substrates, including AR [40]. The dynamic spatial co-localization patterns of AR (cytoplasmic in absence of androgen, nuclear upon stimulation) [41] and DDB1 (cytoplasmic, translocating to nucleus upon UV exposure) [23] further support their potential for functional interaction.

RBM15 modulates mRNA expression via m6A modifications, a principle demonstrated across various cancers (e.g., upregulating YES1 in HCC [42], enhancing OTUB2 in cervical cancer [43]). In PCa, we found RBM15 increases m6A levels in the CDS region of DDB1 mRNA. This m6A mark is then recognized by YTHDF2, promoting DDB1 mRNA degradation and subsequently decreasing DDB1 protein expression. This mechanism is consistent with other studies where m6A readers like YTHDF2 mediate mRNA decay [44, 45]. Crucially, our results also reveal a reciprocal regulatory feedback loop: AR signaling pathway regulates RBM15. Androgen stimulation or AR overexpression upregulated RBM15. Clinically, short-term androgen deprivation decreased RBM15, aligning with initial ADT efficacy. However, long-term deprivation, mirroring CRPC development, increased RBM15. This suggests AR pathway alterations, like frequent AR gene amplification in CRPC [46], may drive elevated RBM15 expression in castration-resistant states. Thus, RBM15 actively participates in CRPC progression, governed by this complex AR feedback.

Our study has certain limitations. First, the precise molecular mechanisms by which AR signaling regulates RBM15 expression require further elucidation. Investigating the transcriptional or post-transcriptional control of RBM15 by AR would provide a more complete understanding of this feedback loop. Second, independent validation through clinical cohort follow-up data to prove RBM15’s role in PCa progression is still needed. Such studies would strengthen the generalizability and clinical applicability of RBM15 as a prognostic biomarker.

In summary, this study identifies RBM15 as a key m6A methyltransferase component promoting PCa progression and poor prognosis, establishing it as a novel therapeutic target. We uncovered a positive feedback loop: RBM15-mediated m6A modification decreases DDB1, leading to reduced AR ubiquitination and degradation. Reciprocally, AR signaling enhances RBM15 expression. This intricate crosstalk between RNA modification and protein post-translational modification offers a new molecular paradigm for CRPC progression. Co-targeting RBM15 and AR represents a promising strategy to overcome ADT resistance in PCa.

## Materials and methods

### Main reagents

RPMI-1640 medium was purchased from Boster Biological Technology Co., Ltd. (Wuhan, China). Enzalutamide was obtained from MedChemExpress (MCE).

The proteasome inhibitor MG132, protein synthesis inhibitor cycloheximide (CHX), and m6A methylation inhibitor 3-deazaadenosine (DAA) were also sourced from MCE (New Jersey, USA). The lysosomal inhibitor chloroquine (CQ) was obtained from Selleck (Houston, USA), and the DNA transcription inhibitor actinomycin D was purchased from Cayman Chemical (Ann Arbor, USA).

### Cell lines and cell culture

The human prostate cancer cells used in this study included the 22Rv1, LNCaP and C4-2B EnzR cell lines, which were obtained from American Type Culture Collection (Manassas, VA, USA). As described in our previous study [21], C4-2B EnzR cell lines are resistant to enzalutamide and were derived from C4-2B clones that survived treatment with 20 μM enzalutamide. RPMI-1640 complete medium containing 10% fetal bovine serum (FBS) (ExCell Bio, Suzhou, China) was utilized to culture the cells. The cells were incubated at 37 °C with 5% CO_2_.

### Generation of stable cell lines

Lentivirus shRNA targeting RBM15 and negative controls, as well as lentiviruses expressing RBM15, were designed and developed by Genomeditech (Shanghai, China). C4-2B EnzR cells were transfected with lentivirus-shRNA to stably knockdown RBM15. 22Rv1 cells were transfected with lentivirus expressing RBM15 for stable overexpression. After 72 h of transfection, the cells were treated with puromycin to select stable cell lines.

### siRNA and plasmid transfection

siRNAs targeting RBM15, DDB1, YTHDF2, AR, and their corresponding negative controls were designed and synthesized by Genomeditech (Shanghai, China) or General Biol (Anhui, China). The siRNA sequences are presented in Supplementary Table S3. The Flag-RBM15, MYC-AR, Flag-DDB1, and HA-Ub-K48 plasmids were purchased from Miaoling Biology (Wuhan, China). The DDB1 promoter sequence was cloned and inserted into the P38699 pMCS-Fluc-SV40-hRluc-Neo vector. The HA-AR plasmid was obtained from Genomeditech. The pmirGLO luciferase vector was employed to insert the wild-type or mutated DDB1-CDS sequence. The wild-type and mutated DDB1 CDS reporters were constructed by Gene Create (Wuhan, China). All the siRNAs and plasmids were transfected into cells via Lipofectamine 3000 (Invitrogen, USA).

### Animal experiments

All male BALB/c nude mice used in this study were purchased from Beijing Vital River Laboratory Animal Technology Co., Ltd. Male nude mice, aged four weeks, were randomly assigned to four groups and subcutaneously injected in the right flank with either C4-2B EnzR cells featuring stable RBM15 knockdown or 22Rv1 cells with stable RBM15 overexpression at approximately 1 ~ 2 × 10^6^ cells per mouse to establish a xenograft tumor model. Seven days post-injection, the tumor sizes were measured every four days using calipers. The volume of each tumor was calculated as width^^2^ × length × 0.5. After the mice were sacrificed, the xenograft tumors were removed, weighed, and photographed. Moreover, the harvested tumors were fixed with 4% paraformaldehyde and embedded in paraffin for further analysis. The animal experiments in this study were approved by the Laboratory Animal Welfare & Ethics Committee of Tongji Hospital of Huazhong university of Science and Technology (TJH-202310015).

### RNA extraction and reverse transcription‒quantitative PCR (RT‒qPCR)

RNA was extracted from cells using the SteadyPure RNA Extraction Kit (Accurate Biology, Changsha, China) according to the manufacturer’s instructions. The extracted RNA was treated with reagents from the Hiscript II Q RT SuperMix for qPCR (+ gDNA wiper) Kit (Vazyme, Nanjing, China) to remove genomic DNA and reverse transcribe it into cDNA. The qPCR mixture was subsequently prepared according to the protocol of the Taq Pro Universal SYBR qPCR Master Mix (Vazyme, Nanjing, China), and the program for the qPCR was set. The primer sequences utilized in the present study are listed in Supplementary Table S4.

### Western blotting

The cells were lysed using RIPA lysis buffer supplemented with 1% PMSF and phosphatase inhibitors, followed by sonication and centrifugation to extract the cellular proteins. The concentration of total protein was determined, and 20 μg was used for electrophoresis. After electrophoresis, the proteins were transferred to a PVDF membrane, which was then blocked with nonfat milk and incubated with primary and secondary antibodies. Following incubation with a Chemiluminescence (ECL) Kit (Servicebio, Wuhan, China), proteins were detected by the ChemiDocTM MP Imaging System (Bio-Rad). The antibodies used for western blotting are listed in Supplementary Table S5.

### RNA sequencing

22Rv1 cells were transfected with plasmids overexpressing RBM15 or with empty vector plasmids. After 72 h of transfection, the cells were harvested via the RNAiso Plus reagent (Takara, Dalian, China) and sent to Gene Read Biotech Co., Ltd. (Wuhan, China) for RNA extraction and detection of the RNA expression profile. Significantly differentially expressed genes (DEGs) were defined as those with an absolute log2-fold change greater than 0.5 and a P value less than 0.05. GO enrichment analysis was subsequently conducted on these DEGs, and terms related to biological process (BP), cellular component (CC), and molecular function (MF) with P value less than 0.05 and ranking in the top 10 terms by count were visualized.

### Coimmunoprecipitation and mass spectrometry analysis

Coimmunoprecipitation experiments were conducted according to the instructions supplied with the Classic Protein A/G Immunoprecipitation Kit (Biolinkedin, Shanghai, China). The cells were lysed and collected using IP lysis/wash buffer supplemented with 1% PMSF and phosphatase inhibitors. The lysates were then divided into input, IgG, and IP groups, and the corresponding antibodies were added to the IgG and IP groups and incubated overnight to form antigen‒antibody complexes. Subsequently, the magnetic stand and protein A/G magnetic beads were used to capture the complexes and separate the unbound components. The captured complexes were then sent to SpecAlly Life Technology Co., Ltd. (Wuhan, China) for mass spectrometry analysis or subjected to western blot analysis.

### Immunofluorescence

Cells seeded on coverslips were fixed with 4% paraformaldehyde and permeabilized with 0.5% Triton X-100. The cells were then blocked with goat serum and incubated with primary antibodies and fluorescence-conjugated secondary antibodies. Subsequently, DAPI solution was added to stain the nuclei, and the coverslips were inverted onto microscope slides. Images were randomly captured using a fluorescence microscope or a confocal laser microscope (Nikon, Tokyo, Japan). The antibodies used for the immunofluorescence assays are listed in Supplementary Table S5.

### Dot blot and m6A methylation quantification

RNA was extracted from the cells and serially diluted with RNase-free ddH_2_O. RNA (200, 400, and 800 ng) was then spotted onto a nylon membrane. After crosslinking with ultraviolet light, the membrane was blocked with 5% nonfat milk and incubated with a primary antibody against m6A and a secondary antibody. After incubation with an ECL Kit, m6A-modified RNA was detected by ChemiDocTM MP Imaging System (Bio-Rad). For m6A methylation quantification, the m6A RNA Methylation Quantification Kit (Colorimetric) (Epigentek, NY, USA) was used to measure the levels of m6A in total RNA. Briefly, after incubation with binding solution, RNA was sequentially incubated with capture antibody, detection antibody, and enhancer solution to capture m6A RNA. Subsequently, the developer solution was added to the RNA until the solution in the positive control turned blue, at which point stop solution was added to terminate the reaction. The absorbance of the solution at 450 nm was measured using a microplate reader.

### RNA immunoprecipitation (RIP)-qPCR and Methylate RNA immunoprecipitation (MeRIP)-qPCR

The RNA immunoprecipitation (RIP) experiment was conducted according to the protocol of PureBinding RNA Immunoprecipitation Kit (GENESEED, Guangzhou, China). The cells were lysed via buffer A containing protease and RNase inhibitors. The cell lysate supernatant was then divided into input, IgG, and IP groups. Following pretreatment with buffer A and buffer D solutions, magnetic beads were conjugated with IgG/IP antibodies. The antibody-coated magnetic beads were added to the corresponding lysate supernatants for immunoprecipitation of proteins and RNA. Subsequently, proteins and RNA were extracted for western blot and RT‒qPCR analysis, respectively. The EpiQuik™ CUT&RUN m6A RNA Enrichment (MeRIP) Kit (Epigentek, NY, USA) was used to carry out the MeRIP experiment. The extracted RNA samples were separated into m6A and IgG groups, and then mixed with immune capture buffer, magnetic beads, and m6A or IgG antibodies to facilitate antibody binding to the beads and capture RNA. The primers utilized for this analysis are shown in Supplementary Table S4.

### RNA pulldown assay

Biotin-labeled ssRNAs with or without m6A modification designed for DDB1 site 1, along with complementary ssDNA, were synthesized via Accurate Biology (Changsha, China). The sequence of the unmodified ssRNA is 5’- UGGCCACUGCGGUCUGACCCUAAUCGUGAGACUG-3’, and the m6A-modified ssRNA sequence is 5’- UGGCCACUGCGGUCUG (m6A) CCCUAAUCGUGAGACUG-3’. The complementary ssDNA sequence is 5’-CAGTCTCACGATTAGGGTCAGACCGCAGTGGCCA-3’. The negative control is poly(A)_25_ RNA. Proteins that bind to DDB1 site 1 were pulled down via an RNA pulldown kit (IEMed, Guangzhou, China). Cells were lysed using Biotin pulldown buffer containing RNase and protease inhibitors, followed by ultrasonication. After the RNA probe’s secondary structure was formed, magnetic beads were added to incubate with the probe and then introduced into the cell lysate supernatant to capture probe-bound proteins. The proteins were subsequently eluted for western blot analysis.

### Statistical analysis

All the data are presented as the means ± standard deviations (SDs). Comparisons between two groups were performed using the independent t test or Mann‒Whitney U test. Multiple groups of data involving more than two groups were compared using one-way ANOVA or the Kruskal‒Wallis H test. The log-rank test was employed to compare differences in survival curves. Statistical analyses were conducted using R software (version 4.2.1) and GraphPad Prism (version 8.0.2). A *P*-value of less than 0.05 was considered to indicate statistical significance.

## Supplementary Information

Supplementary Material 1.

## Data Availability

The RNA-sequecing data generated in this study has been deposited in ScienceDB database (CSTR: 31,253.11.sciencedb.28570), which are publicly accessible at https://www.scidb.cn/. The data utilized in this study are available from the corresponding author upon reasonable request.

## Abbreviations

ADT: Androgen deprivation therapy
AR: Androgen receptor
CHX: Cycloheximide
CQ: Chloroquine
CRPC: Castration-resistant prostate cancer
CS-FBS: Charcoal-stripped fetal bovine serum
DAA: 3-Deazaadenosine
DDB1: Damage-specific DNA binding protein 1
DEG: Differentially expressed gene
DHT: Dihydrotestosterone
EMT: Epithelial-mesenchymal transition
Enz: Enzalutamide
FBS: Fetal bovine serum
GEO: Gene Expression Omnibus
GO: Gene Ontology
KEGG: Kyoto Encyclopedia of Genes and Genomes
m6A: N6-methyladenosine
MeRIP: Methylate RNA immunoprecipitation
MR: Mendelian randomization
PCa: Prostate cancer
RBM15: RNA-binding motif protein 15
RIP: RNA immunoprecipitation
ROC: Receiver operating characteristic
SD: Standard deviation
UPS: Ubiquitin-proteasome system

## References

[1] Bray F, Laversanne M, Sung H, Ferlay J, Siegel RL, Soerjomataram I, et al. Global cancer statistics 2022: GLOBOCAN estimates of incidence and mortality worldwide for 36 cancers in 185 countries. CA Cancer J Clin. 2024;74(3):229–63. 10.3322/caac.21834. 38572751 10.3322/caac.21834

[2] Dai C, Dehm SM, Sharifi N. Targeting the androgen signaling axis in prostate cancer. J Clin Oncol. 2023;41(26):4267–78. 10.1200/JCO.23.00433. 37429011 10.1200/JCO.23.00433 PMC10852396

[3] Siegel RL, Giaquinto AN, Jemal A. Cancer statistics, 2024. CA Cancer J Clin. 2024;74(1):12–49. 10.3322/caac.21820. 38230766 10.3322/caac.21820

[4] Daniels VA, Luo J, Paller CJ, Kanayama M. Therapeutic approaches to targeting androgen receptor splice variants. Cells. 2024. 10.3390/cells13010104. 38201308 10.3390/cells13010104 PMC10778271

[5] Patil DP, Pickering BF, Jaffrey SR. Reading m(6)A in the transcriptome: m(6)A-binding proteins. Trends Cell Biol. 2018;28(2):113–27. 10.1016/j.tcb.2017.10.001. 29103884 10.1016/j.tcb.2017.10.001 PMC5794650

[6] Boulias K, Greer EL. Biological roles of adenine methylation in RNA. Nat Rev Genet. 2023;24(3):143–60. 10.1038/s41576-022-00534-0. 36261710 10.1038/s41576-022-00534-0 PMC9974562

[7] Fang Z, Mei W, Qu C, Lu J, Shang L, Cao F, et al. Role of m6A writers, erasers and readers in cancer. Exp Hematol Oncol. 2022;11(1):45. 10.1186/s40164-022-00298-7. 35945641 10.1186/s40164-022-00298-7 PMC9361621

[8] Wang Y, Wang Y, Patel H, Chen J, Wang J, Chen ZS, et al. Epigenetic modification of m(6)A regulator proteins in cancer. Mol Cancer. 2023;22(1):102. 10.1186/s12943-023-01810-1. 37391814 10.1186/s12943-023-01810-1 PMC10311752

[9] Shi H, Wei J, He C. Where, when, and how: context-dependent functions of RNA methylation writers, readers, and erasers. Mol Cell. 2019;74(4):640–50. 10.1016/j.molcel.2019.04.025. 31100245 10.1016/j.molcel.2019.04.025 PMC6527355

[10] He L, Li H, Wu A, Peng Y, Shu G, Yin G. Functions of N6-methyladenosine and its role in cancer. Mol Cancer. 2019;18(1):176. 10.1186/s12943-019-1109-9. 31801551 10.1186/s12943-019-1109-9 PMC6892141

[11] Li X, Ma S, Deng Y, Yi P, Yu J. Targeting the RNA m(6)A modification for cancer immunotherapy. Mol Cancer. 2022;21(1):76. 10.1186/s12943-022-01558-0. 35296338 10.1186/s12943-022-01558-0 PMC8924732

[12] Liu Z, Zou H, Dang Q, Xu H, Liu L, Zhang Y, et al. Biological and pharmacological roles of m(6)A modifications in cancer drug resistance. Mol Cancer. 2022;21(1):220. 10.1186/s12943-022-01680-z. 36517820 10.1186/s12943-022-01680-z PMC9749187

[13] Diao MN, Zhang XJ, Zhang YF. The critical roles of m6A RNA methylation in lung cancer: from mechanism to prognosis and therapy. Br J Cancer. 2023;129(1):8–23. 10.1038/s41416-023-02246-6. 36997662 10.1038/s41416-023-02246-6 PMC10307841

[14] Ding SQ, Zhang XP, Pei JP, Bai X, Ma JJ, Zhang CD, et al. Role of N6-methyladenosine RNA modification in gastric cancer. Cell Death Discov. 2023;9(1):241. 10.1038/s41420-023-01485-z. 37443100 10.1038/s41420-023-01485-z PMC10344904

[15] Auld FM, Sergi CM, Leng R, Shen F. The role of N(6)-methyladenosine in the promotion of hepatoblastoma: a critical review. Cells. 2022. 10.3390/cells11091516. 35563821 10.3390/cells11091516 PMC9101889

[16] Mayday MY, Biancon G, Wei M, Ramirez C, Moratti I, Pintado-Urbanc AP, et al. RBM15-MKL1 fusion protein promotes leukemia via m6A methylation and Wnt pathway activation. Blood. 2025;146(9):1096–109. 10.1182/blood.2024027712. 40435410 10.1182/blood.2024027712 PMC12783520

[17] Zhang J, Wei J, Sun R, Sheng H, Yin K, Pan Y, et al. A lncrna from the FTO locus acts as a suppressor of the m6A writer complex and p53 tumor suppression signaling. Mol Cell. 2023. 10.1016/j.molcel.2023.06.024. 37478845 10.1016/j.molcel.2023.06.024 PMC10427207

[18] Deng X, Su R, Weng H, Huang H, Li Z, Chen J. RNA N(6)-methyladenosine modification in cancers: current status and perspectives. Cell Res. 2018;28(5):507–17. 10.1038/s41422-018-0034-6. 29686311 10.1038/s41422-018-0034-6 PMC5951805

[19] Wang X, Tian L, Li Y, Wang J, Yan B, Yang L, et al. RBM15 facilitates laryngeal squamous cell carcinoma progression by regulating TMBIM6 stability through IGF2BP3 dependent. J Exp Clin Cancer Res. 2021;40(1):80. 10.1186/s13046-021-01871-4. 33637103 10.1186/s13046-021-01871-4 PMC7912894

[20] Park SH, Ju J-S, Woo H, Yun HJ, Lee SB, Kim S-H, et al. The m6A writer RBM15 drives the growth of triple-negative breast cancer cells through the stimulation of serine and glycine metabolism. Exp Mol Med. 2024;56(6):1373–87. 10.1038/s12276-024-01235-w. 38825643 10.1038/s12276-024-01235-w PMC11263342

[21] Hu B, Lin D, Liu Z, Chen R, Liu J, Wu Y, et al. Identification of RBM15 as a prognostic biomarker in prostate cancer involving the regulation of prognostic m6A-related lncRNAs. Eur J Med Res. 2024;29(1):411. 10.1186/s40001-024-02000-5. 39118157 10.1186/s40001-024-02000-5 PMC11312177

[22] Canon J, Osgood T, Olson SH, Saiki AY, Robertson R, Yu D, et al. The MDM2 inhibitor AMG 232 demonstrates robust antitumor efficacy and potentiates the activity of p53-inducing cytotoxic agents. Mol Cancer Ther. 2015;14(3):649–58. 10.1158/1535-7163.MCT-14-0710. 25567130 10.1158/1535-7163.MCT-14-0710

[23] Iovine B, Iannella ML, Bevilacqua MA. Damage-specific DNA binding protein 1 (DDB1): a protein with a wide range of functions. Int J Biochem Cell Biol. 2011;43(12):1664–7. 10.1016/j.biocel.2011.09.001. 21959250 10.1016/j.biocel.2011.09.001

[24] Patil DP, Chen CK, Pickering BF, Chow A, Jackson C, Guttman M, et al. m(6)A RNA methylation promotes XIST-mediated transcriptional repression. Nature. 2016;537(7620):369–73. 10.1038/nature19342. 27602518 10.1038/nature19342 PMC5509218

[25] Ying Y, Wu Y, Zhang F, Tang Y, Yi J, Ma X, et al. Co-transcriptional R-loops-mediated epigenetic regulation drives growth retardation and docetaxel chemosensitivity enhancement in advanced prostate cancer. Mol Cancer. 2024;23(1):79. 10.1186/s12943-024-01994-0. 38658974 10.1186/s12943-024-01994-0 PMC11041046

[26] Tu C, Fiandalo MV, Pop E, Stocking JJ, Azabdaftari G, Li J, et al. Proteomic analysis of charcoal-stripped fetal bovine serum reveals changes in the insulin-like growth factor signaling pathway. J Proteome Res. 2018;17(9):2963–77. 10.1021/acs.jproteome.8b00135. 30014700 10.1021/acs.jproteome.8b00135 PMC10231688

[27] Fontana F, Anselmi M, Limonta P. Molecular mechanisms and genetic alterations in prostate cancer: from diagnosis to targeted therapy. Cancer Lett. 2022;534:215619. 10.1016/j.canlet.2022.215619. 35276289 10.1016/j.canlet.2022.215619

[28] Litwin MS, Tan HJ. The diagnosis and treatment of prostate cancer: a review. JAMA. 2017;317(24):2532–42. 10.1001/jama.2017.7248. 28655021 10.1001/jama.2017.7248

[29] Hiriart E, Gruffat H, Buisson M, Mikaelian I, Keppler S, Meresse P, et al. Interaction of the Epstein-Barr virus mRNA export factor EB2 with human Spen proteins SHARP, OTT1, and a novel member of the family, OTT3, links Spen proteins with splicing regulation and mRNA export. J Biol Chem. 2005;280(44):36935–45. 10.1074/jbc.M501725200. 16129689 10.1074/jbc.M501725200

[30] Dong H, Zhang H, Mao X, Liu S, Xu W, Zhang Y. RBM15 promates the proliferation, migration and invasion of pancreatic cancer cell lines. Cancers (Basel). 2023. 10.3390/cancers15041084. 36831430 10.3390/cancers15041084 PMC9954619

[31] Park SH, Ju JS, Woo H, Yun HJ, Lee SB, Kim SH, et al. The m(6)A writer RBM15 drives the growth of triple-negative breast cancer cells through the stimulation of serine and glycine metabolism. Exp Mol Med. 2024;56(6):1373–87. 10.1038/s12276-024-01235-w. 38825643 10.1038/s12276-024-01235-w PMC11263342

[32] Zhang J, Wei J, Sun R, Sheng H, Yin K, Pan Y, et al. A lncrna from the FTO locus acts as a suppressor of the m(6)A writer complex and p53 tumor suppression signaling. Mol Cell. 2023;83(15):2692-708 e7. 10.1016/j.molcel.2023.06.024. 37478845 10.1016/j.molcel.2023.06.024 PMC10427207

[33] Bungaro M, Buttigliero C, Tucci M. Overcoming the mechanisms of primary and acquired resistance to new generation hormonal therapies in advanced prostate cancer: focus on androgen receptor independent pathways. Cancer Drug Resist. 2020;3(4):726–41. 10.20517/cdr.2020.42. 35582226 10.20517/cdr.2020.42 PMC8992570

[34] Spataro V, Norbury C, Harris AL. The ubiquitin-proteasome pathway in cancer. Br J Cancer. 1998;77(3):448–55. 10.1038/bjc.1998.71. 9472642 10.1038/bjc.1998.71 PMC2151296

[35] Grice GL, Nathan JA. The recognition of ubiquitinated proteins by the proteasome. Cell Mol Life Sci. 2016;73(18):3497–506. 10.1007/s00018-016-2255-5. 27137187 10.1007/s00018-016-2255-5 PMC4980412

[36] Sharma A, Khan H, Singh TG, Grewal AK, Najda A, Kawecka-Radomska M, et al. Pharmacological modulation of ubiquitin-proteasome pathways in oncogenic signaling. Int J Mol Sci. 2021. 10.3390/ijms222111971. 34769401 10.3390/ijms222111971 PMC8584958

[37] Pham MM, Ngoi NYL, Peng G, Tan DSP, Yap TA. Development of poly(ADP-ribose) polymerase inhibitor and immunotherapy combinations: progress, pitfalls, and promises. Trends Cancer. 2021;7(10):958–70. 10.1016/j.trecan.2021.05.004. 34158277 10.1016/j.trecan.2021.05.004 PMC8458234

[38] Zhang H, Jin X, Huang H. Deregulation of SPOP in cancer. Cancer Res. 2023;83(4):489–99. 10.1158/0008-5472.CAN-22-2801. 36512624 10.1158/0008-5472.CAN-22-2801

[39] Jackson S, Xiong Y. CRL4s: the CUL4-RING E3 ubiquitin ligases. Trends Biochem Sci. 2009;34(11):562–70. 10.1016/j.tibs.2009.07.002. 19818632 10.1016/j.tibs.2009.07.002 PMC2783741

[40] Meyers M, Cismoski S, Panidapu A, Chie-Leon B, Nomura DK. Targeted protein degradation through recruitment of the CUL4 complex adaptor protein DDB1. ACS Chem Biol. 2024;19(1):58–68. 10.1021/acschembio.3c00487. 38192078 10.1021/acschembio.3c00487 PMC11003717

[41] Tan MH, Li J, Xu HE, Melcher K, Yong EL. Androgen receptor: structure, role in prostate cancer and drug discovery. Acta Pharmacol Sin. 2015;36(1):3–23. 10.1038/aps.2014.18. 24909511 10.1038/aps.2014.18 PMC4571323

[42] Cai X, Chen Y, Man D, Yang B, Feng X, Zhang D, et al. RBM15 promotes hepatocellular carcinoma progression by regulating N6-methyladenosine modification of YES1 mRNA in an IGF2BP1-dependent manner. Cell Death Discov. 2021;7(1):315. 10.1038/s41420-021-00703-w. 34707107 10.1038/s41420-021-00703-w PMC8551180

[43] Song Y, Wu Q. RBM15 m(6) A modification-mediated OTUB2 upregulation promotes cervical cancer progression via the AKT/mTOR signaling. Environ Toxicol. 2023;38(9):2155–64. 10.1002/tox.23852. 37334762 10.1002/tox.23852

[44] Chen Y, Pan C, Wang X, Xu D, Ma Y, Hu J, et al. Silencing of METTL3 effectively hinders invasion and metastasis of prostate cancer cells. Theranostics. 2021;11(16):7640–57. 10.7150/thno.61178. 34335955 10.7150/thno.61178 PMC8315076

[45] Yu J, Li W, Hou GJ, Sun DP, Yang Y, Yuan SX, et al. Circular RNA cFAM210A, degradable by HBx, inhibits HCC tumorigenesis by suppressing YBX1 transactivation. Exp Mol Med. 2023;55(11):2390–401. 10.1038/s12276-023-01108-8. 37907737 10.1038/s12276-023-01108-8 PMC10689457

[46] Quigley DA, Dang HX, Zhao SG, Lloyd P, Aggarwal R, Alumkal JJ, et al. Genomic hallmarks and structural variation in metastatic prostate cancer. Cell. 2018;174(3):758-69 e9. 10.1016/j.cell.2018.06.039. 30033370 10.1016/j.cell.2018.06.039 PMC6425931

