# Supplementary material for: Targeting the RNA-binding motif protein 15 suppresses prostate cancer progression and hormone therapy resistance by promoting androgen receptor degradation
Source: Mol Biomed. 2026 Apr 8;7:47. doi: 10.1186/s43556-026-00428-1 (PMC13062041; doi:10.1186/s43556-026-00428-1)
Supplement: Supplementary file 1 — Supplementary Material 1. [file 43556_2026_428_MOESM1_ESM.docx]

**Targeting the RNA-binding motif protein 15 suppresses prostate cancer progression and hormone therapy resistance by promoting androgen receptor degradation**

Bintao Hu 1,2#, Le Li 1#*, Zhenghui Jin 1, Qinyu Li 1, Yue Wu 1, Jie Chen 2, Jihong Liu 1, Chenglin Han 3*, Tao Wang 1*

1 Department of Urology, Tongji Hospital, Tongji Medical College, Huazhong University of Science and Technology, Wuhan, 430030, China.

2 Department of Urology, Jiangxi Provincial People's Hospital, Nanchang, 330000, China.

3 Department of Urology, The First Affiliated Hospital of Shandong First Medical University & Shandong Provincial Qianfoshan Hospital, Jinan, 250000, China.

# These authors contributed equally.

* Corresponding author: Tao Wang, Department of Urology, Tongji Hospital, Tongji Medical College, Huazhong University of Science and Technology, Wuhan, 430030, China. E-mail: [tjhwt@126.com](mailto:tjhwt@126.com). Phone number: +86-17370237995; Chenglin Han, Department of Urology, The First Affiliated Hospital of Shandong First Medical University & Shandong Provincial Qianfoshan Hospital, Jinan, 250000, China. E-mail: [3328@sdhospital.com.cn](mailto:3328@sdhospital.com.cn); Le Li, Department of Urology, Tongji Hospital, Tongji Medical College, Huazhong University of Science and Technology, Wuhan, 430030, China. E-mail: [tjlile2013@163.com](mailto:tjlile2013@163.com).

**Supplementary Figures**


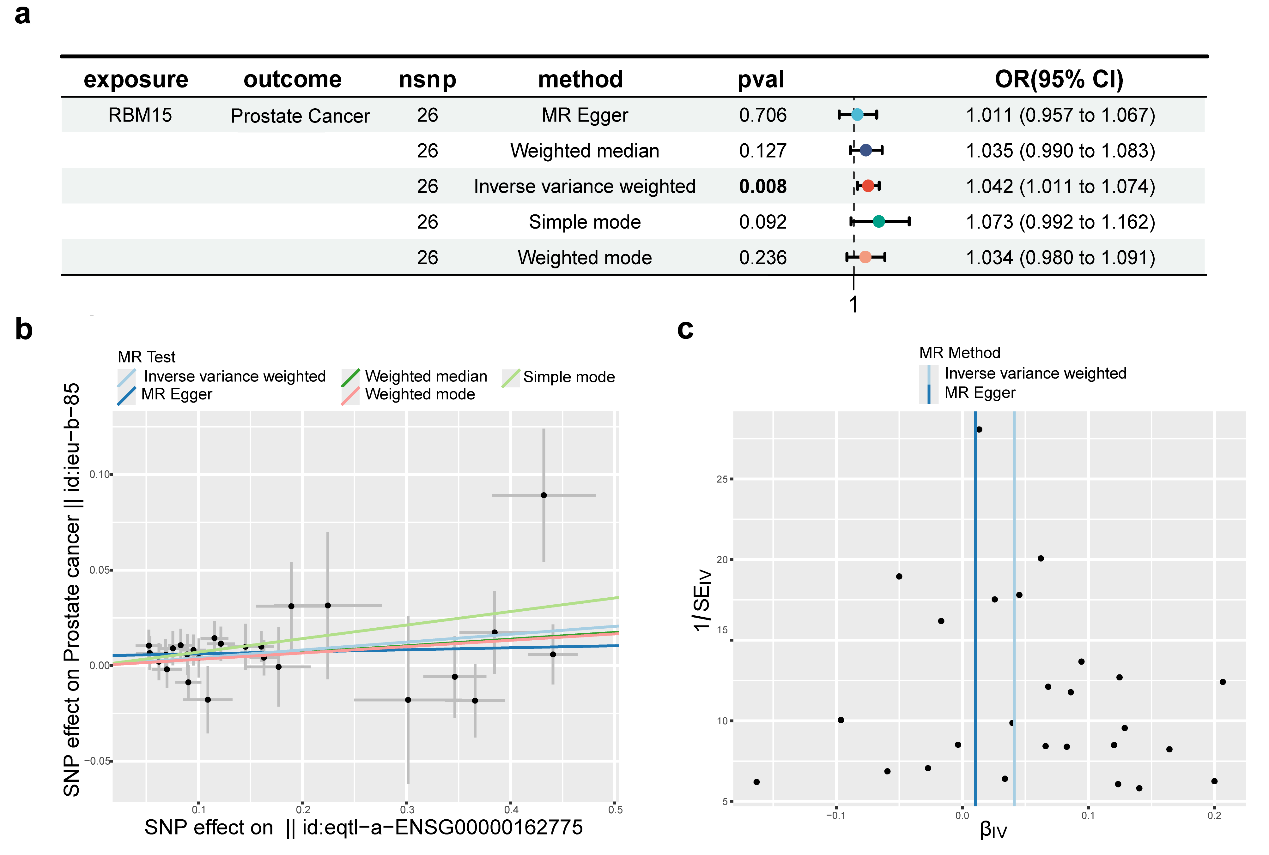


**Supplementary Fig. 1 Visualization of Mendelian randomization analysis. a** Two-sample mendelian randomization analysis for the causal relationship between RBM15 and PCa.  **b** Scatter plot showing the association between RBM15 and prostate cancer. c Funnel plot presenting the heterogeneity analysis of MR.


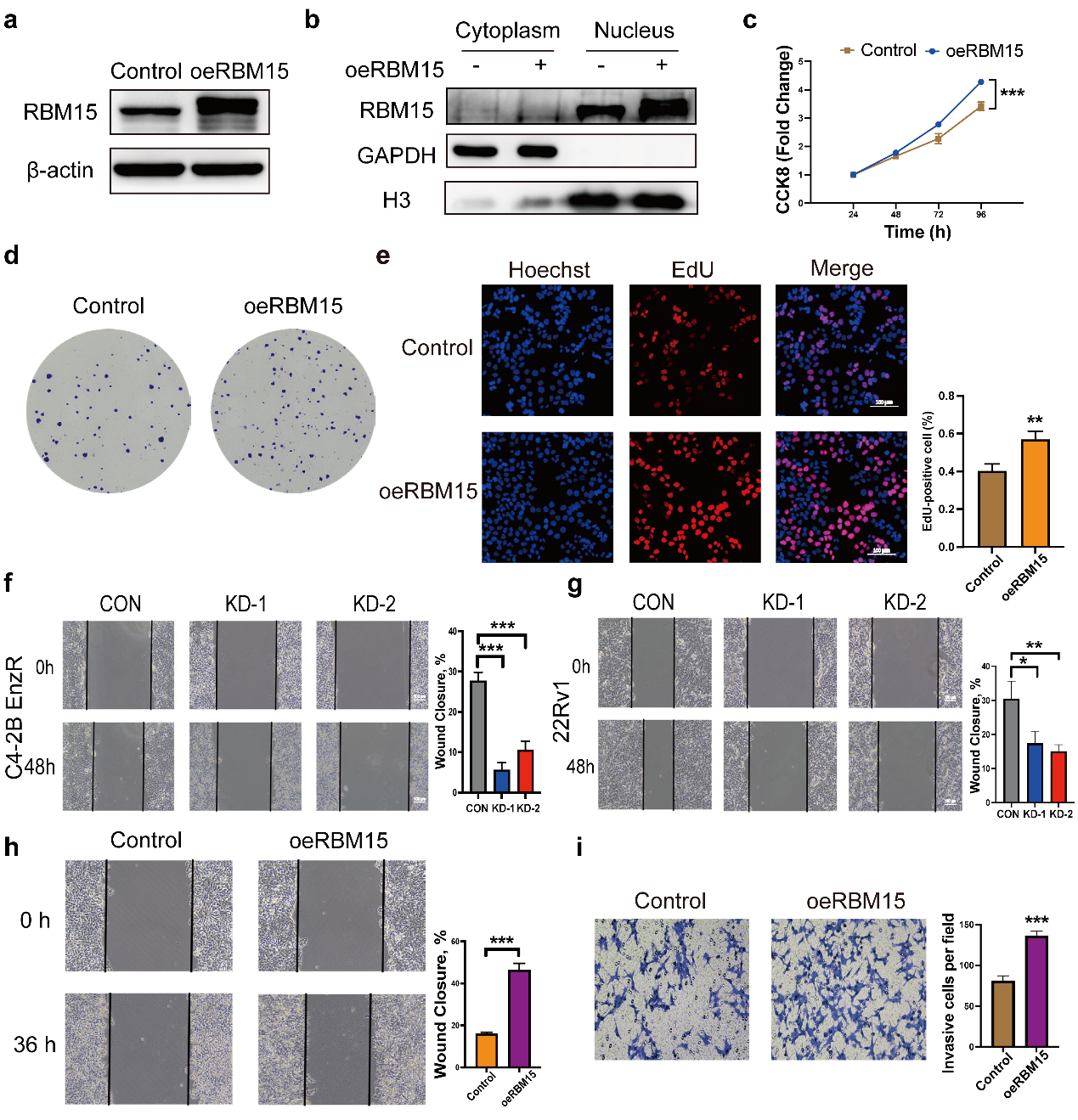


**Supplementary Fig. 2 Overexpression of RBM15 promotes proliferation, migration, and invasion in 22Rv1 cells. a** RBM15 was stably overexpressed in 22Rv1 via lentiviral infection, and the extent of overexpression was measured by western blot. **b** Nuclear-cytoplasmic fractionation assays were used to assess changes in the intracellular distribution of RBM15 upon its overexpression. **c** The impact of RBM15 overexpression on cell viability was determined using the CCK-8 method. **d-e** Colony formation assays (d) and EdU experiments (e) were used to investigate the effects of RBM15 overexpression on the proliferative capacity of cells. **f-g** Changes in the migration and invasion capabilities of cells following RBM15 overexpression were assessed using wound healing assay (f) and transwell invasion assay (g), respectively. All data are presented as mean ± SD. One-way Anova test.


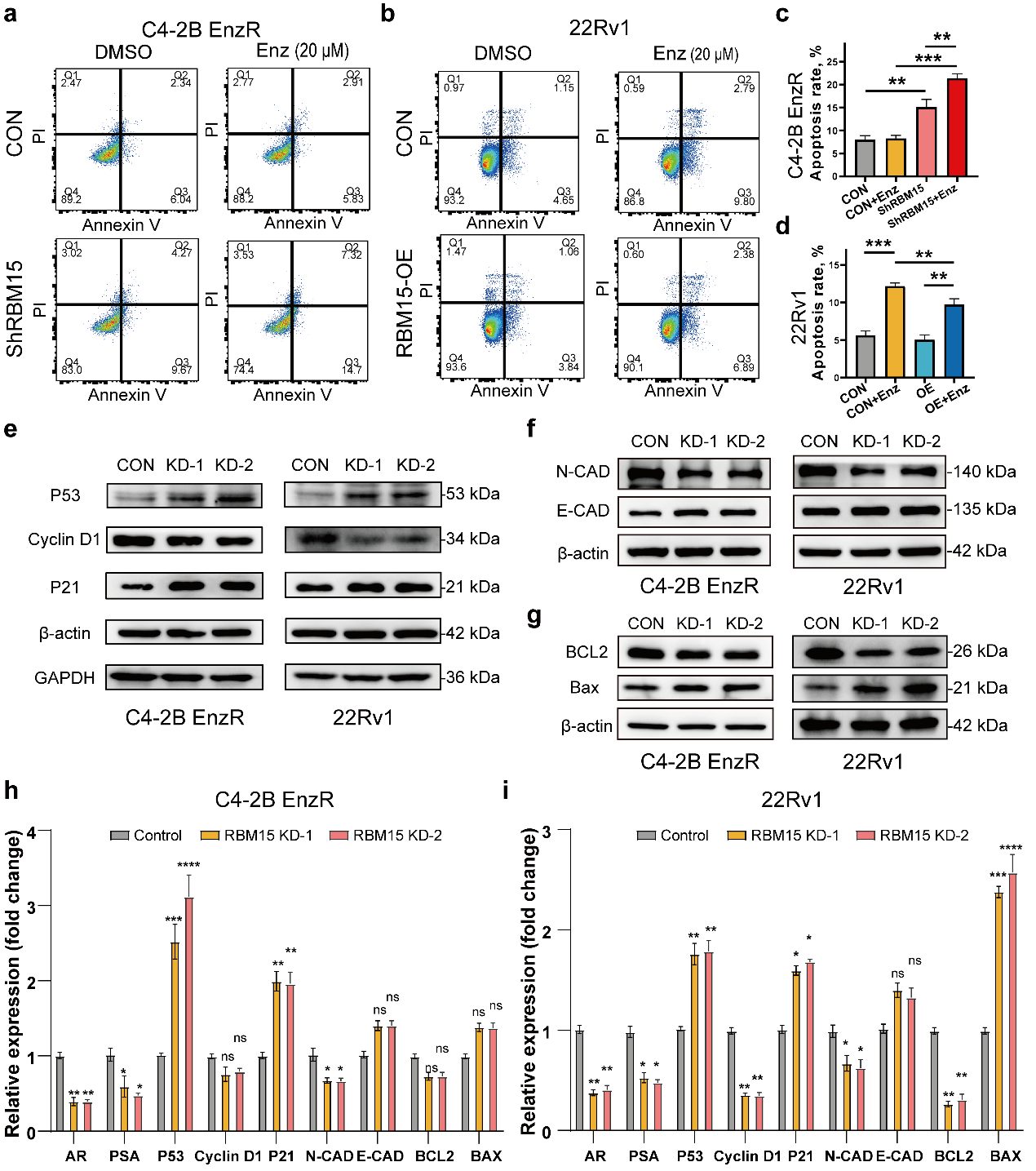


**Supplementary Fig. 3 The impact of RBM15 knockdown on proteins related to the cell cycle, migration and invasion, and apoptosis in C4-2B EnzR and 22Rv1 cells. a-b** Flow cytometry for apoptosis of C4-2B EnzR (a) and 22RV-1 (b) cells showing the impact of RBM15 knockdown or overexpression on the promotion of apoptosis by enzalutamide. **c-d** Statistical analysis for the impact of RBM15 on cell apoptosis induced by enzalutamide. **e** Western blot assay was employed to evaluate the impact of RBM15 silencing on the expression of proteins involved in the P53 signaling pathway. **f** Effects of silencing RBM15 on the expression of EMT-related proteins were examined. **g** Downregulation of RBM15 affected the expression of apoptosis-related proteins. CON represents the control group.

(f) RBM15 on the suppression of cell viability by enzalutamide. **h-i** Statistical analysis for effects of silencing RBM15 on the expression of certain proteins. Two-tailed Student’s t test.


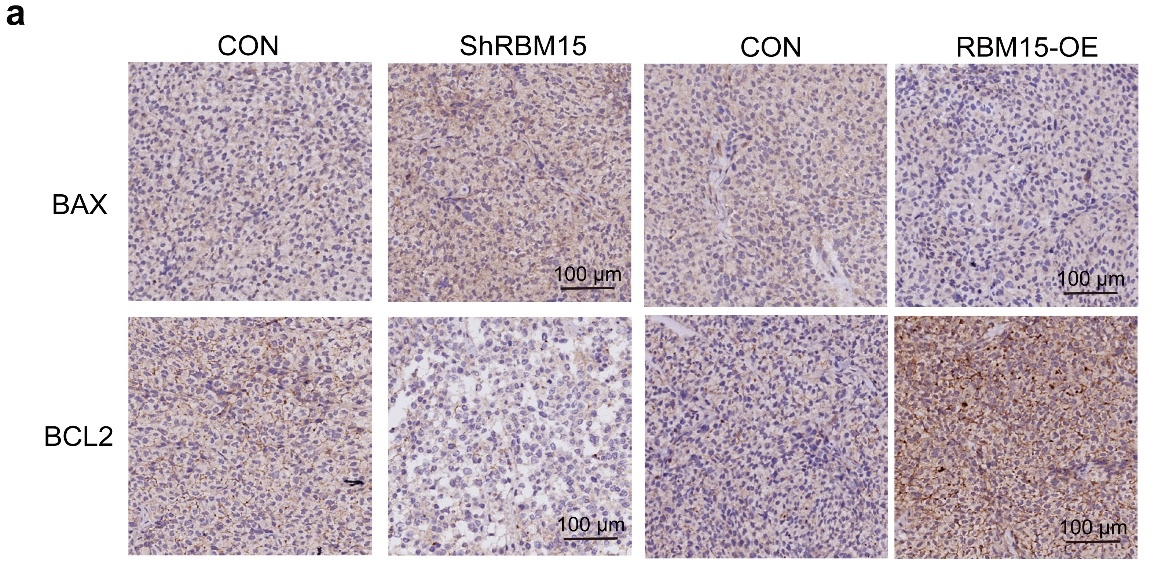


**Supplementary Fig. 4 a** Immunohistochemistry in xenograft tumor tissues reveals the impact of knocking down or overexpressing RBM15 on the expression of apoptosis-related proteins BAX and BCL2. CON denotes the corresponding control group. KD, OE, and CON represent RBM15 knockdown, RBM15 overexpression, and the corresponding control group, respectively.


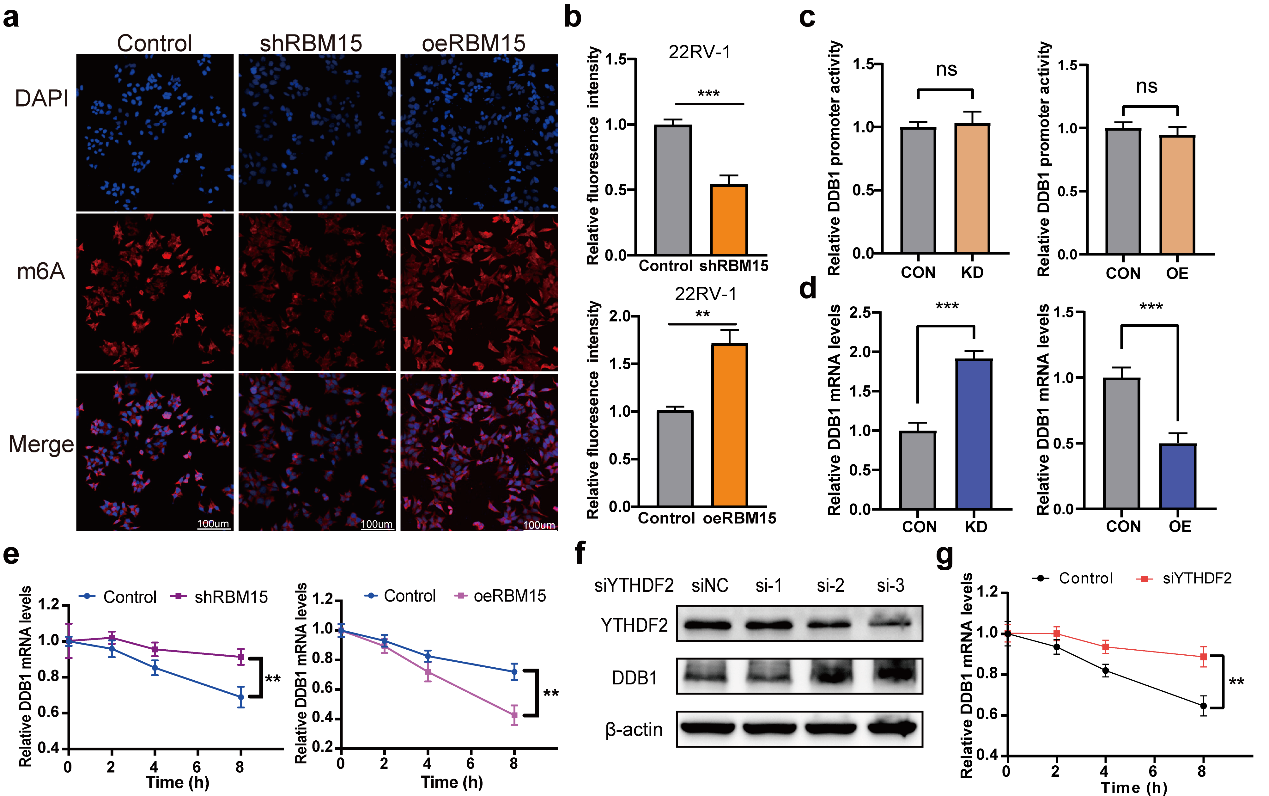


**Supplementary Fig. 5 The effect of YTHDF2 knockdown on DDB1 expression.** **a-b** Immunofluorescence experiments revealing the effect of knocking down or overexpressing RBM15 on intracellular m6A modification levels in 22RV-1 cells. **c** Luciferase reporter assay was used to examine the effects of knocking down or overexpressing RBM15 on DDB1 promoter activity. **d** Cells with RBM15 knockdown or overexpression were treated with 5 μg/mL actinomycin D, followed by measuring the decay rates of DDB1 mRNA. **e** RIP-qPCR with anit-RBM15 antibody and agarose electrophoresis analysis revealed the interaction between RBM15 protein and DDB1 mRNA. **f** Western blotting showed the impact of YTHDF2 knockdown by siRNAs targeting YTHDF2 on YTHDF2 and DDB1 protein levels. **g** The impact of YTHDF2 knockdown on the decay rates of DDB1 mRNA. Two-tailed Student’s t test.

**Supplementary Tables**

| siRNA (human) | Sense 5'-3' | antisense 5'-3' |
| --- | --- | --- |
| siRBM15-1 | ACGAGAAUUUGAUCGAUUU | AAAUCGAUCAAAUUCUCGU |
| siRBM15-2 | GGACAGAGGUGAUCGAGAU | AUCUCGAUCACCUCUGUCC |
| siRBM15-3 | GAAGAUAGAAGCUGUGUAU | AUACACAGCUUCUAUCUUC |
| negative control | UUCUCCGAACGUGUCACGU | ACGUGACACGUUCGGAGAA |
| siAR-1 | CGUCUACCCUGUCUCUCUATT | UAGAGAGACAGGGUAGACGTT |
| siAR-2 | GCUGCAAGGUCUUCUUCAATT | UUGAAGAAGACCUUGCAGCTT |
| siAR-3 | GAUCCUUCACCAAUGUCAATT | UUGACAUUGGUGAAGGAUCTT |
| negative control | UUCUCCGAACGUGUCACGUTT | ACGUGACACGUUCGGAGAATT |
| siDDB1-1 | UGAUAAUGGUGUUGUGUUUTT | AAACACAACACCAUUAUCATT |
| siDDB1-2 | CUGAAGAGGCAGAGCCCAATT | UUGGGCUCUGCCUCUUCAGTT |
| siDDB1-3 | AGAGAUUGCUCGAGACUUUTT | AAAGUCUCGAGCAAUCUCUTT |
| negative control | UUCUCCGAACGUGUCACGUTT | ACGUGACACGUUCGGAGAATT |
| siYTHDF2-1 | CAUGAAUACUAUAGACCAATT | UUGGUCUAUAGUAUUCAUGTT |
| siYTHDF2-2 | GAUAUUGGAACUUGGGAUATT | UAUCCCAAGUUCCAAUAUCTT |
| siYTHDF2-3 | ACGUCAAGGUCGUGGGAAATT | UUUCCCACGACCUUGACGUTT |
| negative control | UUCUCCGAACGUGUCACGUTT | ACGUGACACGUUCGGAGAATT |

**Table S1.** Sequence for siRNAs

| Genes (human) | Forward 5'-3' | Reverse 5'-3' |
| --- | --- | --- |
| DDB1 mRNA | GGGAAGATTGCGGTCATGGA | ATGGGCTCGCGTAATGATGT |
| DDB1 Site 1 | CATGCCAGCATTGACTTACCA | GGTTTCTTCTACCTCCTCTCCAT |
| DDB1 Site 2 | CACACAGAGATGGAACATGAAGT | TCAAGATACGAGCCGAGATGT |
| DDB1 Site 3 | TCGCATTGTGGTCTTTCAGTAT | CGTGCTATTGATGCTGGCTAAC |
| DDB1 Site 4 | GACCTTATGCGCTCAGTGCT | CCAAGATTTCCACAGCACTCATC |
| DDB1 Site 5-6 | CACAGTTCCCCTATGTGGAAGT | GTCTAAGGTGATGGCTGGAATCA |
| β-actin | GTCATTCCAAATATGAGATGCGT | GCTATCACCTCCCCTGTGTG |

**Table S2.** Primer sequences for RT-qPCR analysis

**Table S3.** Antibodies used in this study

| Antibody Name | Antibody Brand | Catalog Number |
| --- | --- | --- |
| anti-RBM15 | Proteintech | 10587-1-AP |
| anti-β-actin | Abclonal | AC026 |
| anti-GAPDH | Boster | A00227-1 |
| anti-Histone H3 | Proteintech | 17168-1-AP |
| anti-AR | Cell signaling technology | 5153s |
| anti-KLK3 | Affinity | AF0246 |
| anti-p53 | Abclonal | A16989 |
| anti-p21 | Abclonal | A19094 |
| anti-Cyclin D1 | Abclonal | A11310 |
| anti-N-Cadherin | Abclonal | A0433 |
| Anti-E-Cadherin | Cell signaling technology | 3195s |
| anti-BAX | Proteintech | 50599-2-Ig |
| anti-BCL2 | Proteintech | 26593-1-AP |
| anti-ubiquitin | Proteintech | 10201-2-AP |
| anti-ubiquitin | Cell signaling technology | 3936 |
| anti-IgG | Abclonal | AC005 |
| HRP Conjugated anti-rabbit IgG | Boster | BA1054 |
| anti-rabbit IgG for IP (HRP) | Vazyme | RA1008-01 |
| anti-DDB1 | Proteintech | 11380-1-AP |
| anti-DDB1 | Affinity | DF7100 |
| anti-HA | Proteintech | 51064-2-AP |
| anti-Flag | Abclonal | AE092 |
| anti-MYC | Proteintech | 60003-2-Ig |
| DyLight 594, anti-Rabbit IgG | Abbkine | KTD109 |
| CoraLite488-conjugated anti-Mouse IgG (H+L) | Proteintech | SA00013-1 |
| Anti-m6A | Abcam | ab284130 |
| Anti-YTHDF2 | Proteintech | 24744-1-AP |

| Exposure | Outcome | Egger_intercept | se | pval |
| --- | --- | --- | --- | --- |
| RBM15 \|\| id: eqtl-a-ENSG00000162775 | Prostate cancer \|\| id: ieu-b-85 | 0.005 | 0.004 | 0.193 |

**Table S4.** Pleiotropy analysis of MR

| Exposure | Outcome | Method | Q | Q_df | Q_pval |
| --- | --- | --- | --- | --- | --- |
| RBM15 \|\| id: eqtl-a-ENSG00000162775 | Prostate cancer \|\| id: ieu-b-85 | MR Egger | 17.47 | 24 | 0.83 |
| RBM15 \|\| id: eqtl-a-ENSG00000162775 | Prostate cancer \|\| id: ieu-b-85 | Inverse variance weighted | 19.26 | 25 | 0.78 |

**Table S5.** Heterogeneity analysis of MR
